# Transcriptomic analyses of cacao cell suspensions in light and dark provide target genes for controlled flavonoid production

**DOI:** 10.1038/s41598-018-31965-7

**Published:** 2018-09-11

**Authors:** Adriana M. Gallego, Luisa F. Rojas, Oriana Parra, Héctor A. Rodriguez, Juan C. Mazo Rivas, Aura Inés Urrea, Lucía Atehortúa, Andrew S. Fister, Mark J. Guiltinan, Siela N. Maximova, Natalia Pabón-Mora

**Affiliations:** 1 Universidad de Antioquia, Grupo de Biotecnología, Medellín, Colombia; 2 Universidad de Antioquia, Grupo de Biotecnología-Escuela de Microbiología, Medellín, Colombia; 3 Corporación para Investigaciones Biológicas and Departamento de Ciencias Agronómicas, Facultad de Ciencias Agrarias, Universidad Nacional de Colombia, UNALMED-CIB, Medellín, Colombia; 4 Compañía Nacional de Chocolates, Medellín, Colombia; 5 Department of Plant Science and Huck Institutes of Life Sciences, The Pennsylvania State University, University Park, PA United States; 6 Universidad de Antioquia, Instituto de Biología, Grupo Evo-Devo en Plantas, Medellín, Colombia

## Abstract

Catechins, including catechin (C) and epicatechin (E), are the main type of flavonoids in cacao seeds. They play important roles in plant defense and have been associated with human health benefits. Although flavonoid biosynthesis has been extensively studied using *in vitro* and *in vivo* models, the regulatory mechanisms controlling their accumulation under light/dark conditions remain poorly understood. To identify differences in flavonoid biosynthesis (particularly catechins) under different light treatments, we used cacao cell suspensions exposed to white-blue light and darkness during 14 days. RNA-Seq was applied to evaluate differential gene expression. Our results indicate that light can effectively regulate flavonoid profiles, inducing a faster accumulation of phenolic compounds and shifting E/C ratios, in particular as a response to switching from white to blue light. The results demonstrated that *HY5, MYB12*, *ANR* and *LAR* were differentially regulated under light/dark conditions and could be targeted by overexpression aiming to improve catechin synthesis in cell cultures. In conclusion, our RNA-Seq analysis of cacao cells cultured under different light conditions provides a platform to dissect key aspects into the genetic regulatory network of flavonoids. These light-responsive candidate genes can be used further to modulate the flavonoid production in *in vitro* systems with value-added characteristics.

## Introduction

Cacao seeds are a rich source of polyphenols, particularly flavonoids^1^. Flavonoids play important roles in plant defenses against herbivory^2^, pathogens^3,4^, cold^5^, drought^6^, high light intensity^7^ and UV radiation^8^. These compounds are often produced in response to different stressors^9,10^. The main groups of flavonoids in cacao include catechins (flavan-3-ols) (37%), proanthocyanidins (PAs) (58%) and anthocyanins (4%)^11^. Among the catechins, (−)- epicatechin is more abundant than (+)- catechin, representing 35% of the polyphenol content in unfermented cacao beans^12^. Catechins serve also as monomeric units for the construction of proanthocyanidin oligomers and share the same substrate for the production of anthocyanins^9^. Cacao flavonoids, specifically catechins and their oligomers contribute to the chocolate quality, as they are responsible for the astringency^13^. Additionally, they have been associated with potential benefits for human health^14^, particularly in reducing cardiovascular disease^15^.

Flavonoid production is regulated at the transcriptional level by different families of transcription factors, in particular the multimeric unit formed by members of the R2R3-MYBs, bHLHs and WD40s, called MBW complexes^16,17^. The early biosynthetic steps are transcriptionally regulated by MYB11, MYB12, and MYB111, three closely related R2R3-MYB proteins. Specific combinations of MBW complexes like the MYB protein TRANSPARENT TESTA 2 (TT2), the bHLH protein TT8 (TRANSPARENT TESTA 8) and the WD40 protein TRANSPARENT TESTA GLABROUS1 (TTG1) control the late reactions of the pathway^18,19^. Functional studies of MBW complexes have been done extensively in maize (*Zea mays*), petunia (*Petunia hybrida*), snapdragon (*Antirrhinum majus*), and Arabidopsis (*Arabidopsis thaliana*)^20–24^. In cacao, functional characterization of the putative *MYB TT2-like* has been done by functional complementation of Arabidopsis mutants^25,26^.

MBW complexes can directly activate downstream structural genes to turn on flavonoid biosynthesis^27–29^. Thus, chalcone synthase (*CHS*), chalcone isomerase (*CHI*), flavanone 3 hydroxylase (*F3H*), flavonoid 3′-hydroxylase (*F3*′*H*), flavonoid 3′5′-hydroxylase, and flavanol synthase (FLS) are early genes in the pathway. Downstream, another set of genes including dihydroflavonol 4-reductase (*DFR*), UDP-glucose flavonol 3-O glucosyl transferase (*UFGT*), anthocyanidin synthase (*ANS*), anthocyanidin reductase (*ANR*), and leucoanthocyanidin reductase (*LAR*) participate as late genes in the pathway^30^. The activation of *ANS*, *ANR*, and *LAR* control the production of catechins ((+)- catechin and (−)- epicatechin) in cacao^25^. In addition, transport genes such as Multidrug and Toxic Compound Extrusion (*MATE*) as well as ABC transporters and the glutathione S-transferase (*GST*) factors have been linked to flavonoid transport and accumulation^31^.

The accumulation of flavonoids *in vivo* can be regulated by environmental conditions, including light^32–34^. Light is perceived by plant photoreceptors, which are localized in the cytoplasm and respond to light stimuli by initiating a cascade of effectors, such as GTP binding proteins (G-proteins) and protein kinases^35^. The rapid induction of flavonoid biosynthesis generally observed under high light intensity demonstrates the key role of flavonoids in photoprotection^36^. Whereas in most plants flavonoid biosynthesis is affected by light^33^, there are some examples of flavonoid accumulation in a light independent manner, as is the case of anthocyanin accumulation during ripening of mangosteen (*Garcinia mangostana*)^37^ and the induction of differential flavonoid profiles in panicles of red rice in the dark^38^.

Understanding how light regulates flavonoid biosynthesis *in planta* can be challenging given the lack of full control over environmental variables, the divergent responses of flavonoid accumulation among plant organs, as well as species and even cultivar- specific responses to light^39–41^. Instead, cell cultures provide a powerful platform for assessing genetic changes associated with the production and accumulation of secondary metabolites such as flavonoids under light treatments thanks to characteristics like its uniformity, homogeneity, repeatability, the absence of developmental processes, and slow systemic effects between cells^42^. Cell cultures override most limitations of studying flavonoid accumulation *in planta* and allow to directly test the influence of light in flavonoid production. *In vitro and in vivo*, flavonoids are known to be induced and accumulated by light stress using LED lights in distinct plant species, particularly blue LED light^43–45^. Flavonoids are also known to be produced in the dark, linked to the induction of cell death and the activity of oxidative enzymes^46–49^. Yet, the influence of light on flavonoid accumulation in *in vitro* systems is still poorly understood, even though catechin production is feasible in cacao cell suspensions and provides a harvesting material for flavonoids^47^.

**Figure 1:**
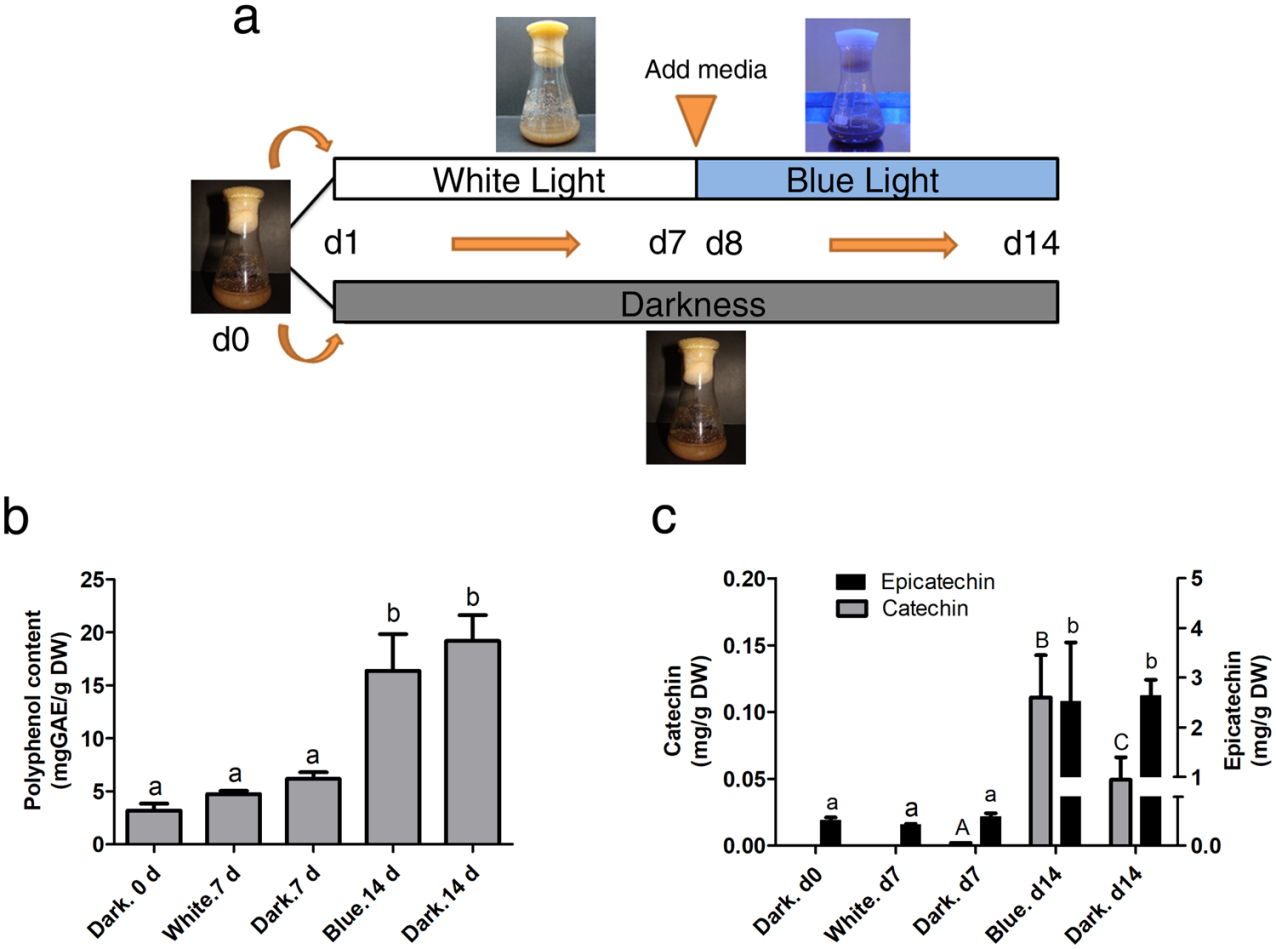
Experimental setup and chemical quantifications for cacao cell suspensions under light treatments and polyphenol content. (**a**) Diagram showing treatments with white - blue LED lights (top) *versus* dark (bottom) along the 14-day time course. (**b**) Measurements of total polyphenol content. (**c**) Measurements of catechin and epicatechin contents during the time course experiment. d: day. The graph shows the average values from three independently sampled cell suspensions. Error bars indicate the standard deviation. Different letters between each treatment indicate p < 0.05.

To better assess the expression changes of genes involved in flavonoid synthesis under light and dark treatments *in vitro*, we sequenced and analyzed 36 transcriptomic profiles of cacao cell suspensions cultured under 9 light and dark treatments. Specifically, cell cultures were subjected to two treatments: (1) white LED light followed by blue LED light (hereafter referred to as W-B) and (2) darkness (hereafter referred to as D) along a time course of 14 days (Fig. 1). Our analysis demonstrated that genes associated with light signaling, transcription factors and structural genes of the flavonoid pathway were differentially expressed in both treatments. Additionally, co-expression networks were built to identify genes associated with the late structural genes in the flavonoid pathway. These results provide additional information in the flavonoid genetic network and suggest candidate genes that serve as targets for metabolic engineering in the production of flavonoids in light/dark conditions.

## Results

### Effect of light treatments on accumulation of total flavonoids in *in vitro* cacao cultures

The accumulation of flavonoids was evaluated in cacao cell suspensions *in vitro* under the two conditions white-blue LED lights (W-B) and dark (D) (Fig. 1a). Considering that flavonoid biosynthesis occurs slowly and dramatic shifts have not been observed after one day^27^, we sampled at three time points corresponding to 0, 7, 8 and 14 days after culture initiation. Compared to day 0 (3.187 mg/g), both W-B and D treatments significantly increased total polyphenol accumulation to 16.39 mg/g and 19.19 mg/g respectively (p < 0.0001) (Fig. 1b). Similarly, total proanthocyanidins (PAs) content showed significant changes from day 0 to day 14. Interestingly, we observed a trend in increasing accumulation of PAs resulting from both light and dark treatments, being higher for light than dark (Supplementary Fig. S1). Epicatechin significantly increased along the time course for both treatments. Catechin content was significantly higher (t-test p < 0.05) in W-B compared to D condition at day 14 (Fig. 1c).

### RNA-Seq analysis and functional annotation

High-throughput sequencing of transcripts from the cell suspensions samples generated 16.91–26.61 million 100-bp single-end reads from each library. After the quality filtering process, 98.90% of the reads remained, with a Q38 percentage ≥80%. The counts of clean reads per library ranged from 16.57 to 26.34 M. The percentages of mapped reads were 43.72–88.49% (Supplementary Table S1). Reads mapped to approximately 17,500 gene models in each library, about 73.93% of the 23,670 genes present in V2 Criollo cacao genome database (http://cocoa-genome-hub.southgreen.fr/). In total, 17,871 known genes were identified, corresponding to 75.50% of cacao genes. GO terms showed that most genes were grouped in cell part (86%) and cell (86%) for the cellular component, cellular process (59%) and metabolic process (51%) for biological process component, and binding (50%) for molecular function (Supplementary Fig. S2). COG analyses identified 3033 genes in general function prediction (R), 1429 in transcription (K) and 347 genes in secondary metabolites biosynthesis, transport and catabolism (Q) (Supplementary Fig. S3).

### Differential gene expression in light versus dark over time

**Figure 2:**
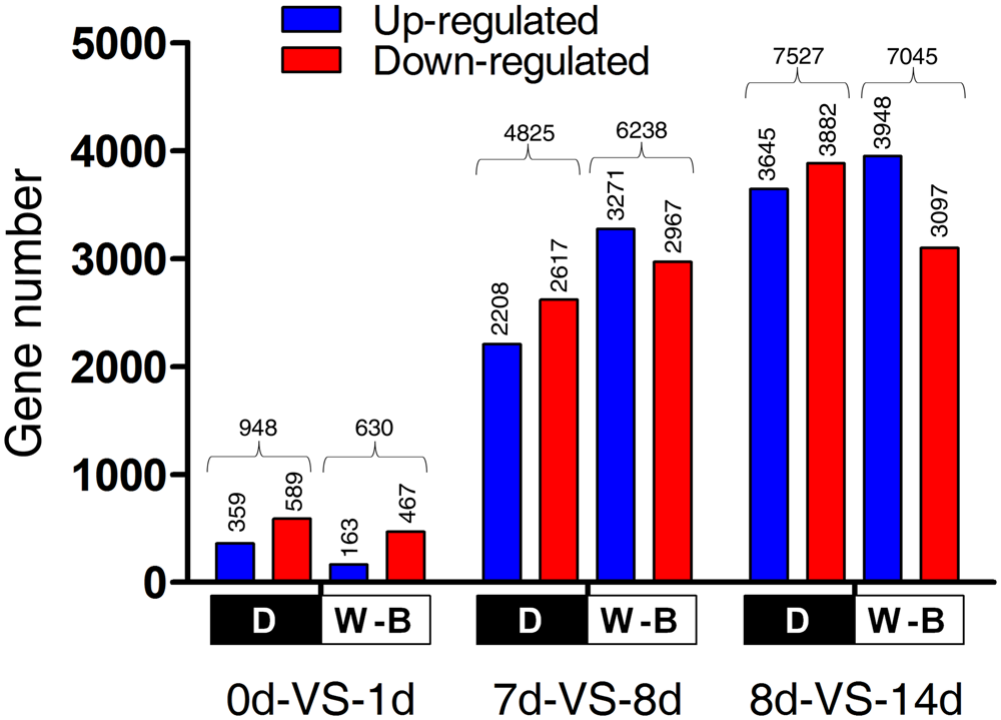
Total number of DEGs in pairwise comparisons over the time course for all cell cultures sequenced under both W-B and D conditions. Values above columns represent total DEGs, up and down regulated genes. d: day.

A total of 10,446 non-redundant differentially expressed genes (DEGs) were identified between W-B and D conditions during the entire time course of the experiment. From these, 6,640 DEGs were found in both treatments, whereas 1,816 DEGs were exclusively expressed in W-B and 1,989 were expressed only in D. In the three pairwise comparisons (by days) we identified 948 (d0-VS-d1), 4,825 (d7-VS-d8) and 7,527 (d8-VS-d14) in D and 630, 6,238 and 7,045 respectively in W-B. We found two opposite trends in gene expression for both treatments. More genes were down-regulated for D (7,088 DEGS) along the progression of the time course compared to W-B where more up-regulated genes (7,382 DEGs), except for the first comparison of day 0 to day 1 (Fig. 2).

Gene ontology analysis for DEGs showed that for the W-B treatment, cellular process, metabolic process, cell part, and cell, constituted the most abundant categories in all the three pairwise comparisons (Supplementary Fig. S4). By contrast, the same analysis in samples subjected to D treatment showed that the gene categories that are more represented include immune system process, growth, cell part and cell (Supplementary Fig. S5).

### Pathway analysis

To determine the effect of W-B and D treatments on gene expression associated to flavonoid production and accumulation in cell cultures, we performed a KEGG enrichment for DEGs in the three pairwise comparisons. KEGG analysis resulted in 66 and 63 pathways for W-B and D, respectively (Supplementary Table S2). For W-B, the most abundant sequences were found in protein processing (38 DEGs, 19.7%) in d0-VS-d1, and metabolic pathways (673, 36.14% and 734 DEGs, 39.41%) for d7-VS-d8 and d8-VS-d14 respectively. In contrast, for the D condition, plant-pathogen interaction (14 DEGs, 11.2%) was enriched at d0-VS-d1 and metabolic pathways (506 DEGs, 27.17% and 706, 37.91%) were enriched for d7-VS-d8 and d8-VS-d14 respectively. Flavonoid biosynthesis was only enriched in the W-B treatment in d7-VS-d8, coinciding with the change from white to blue light.

**Figure 3:**
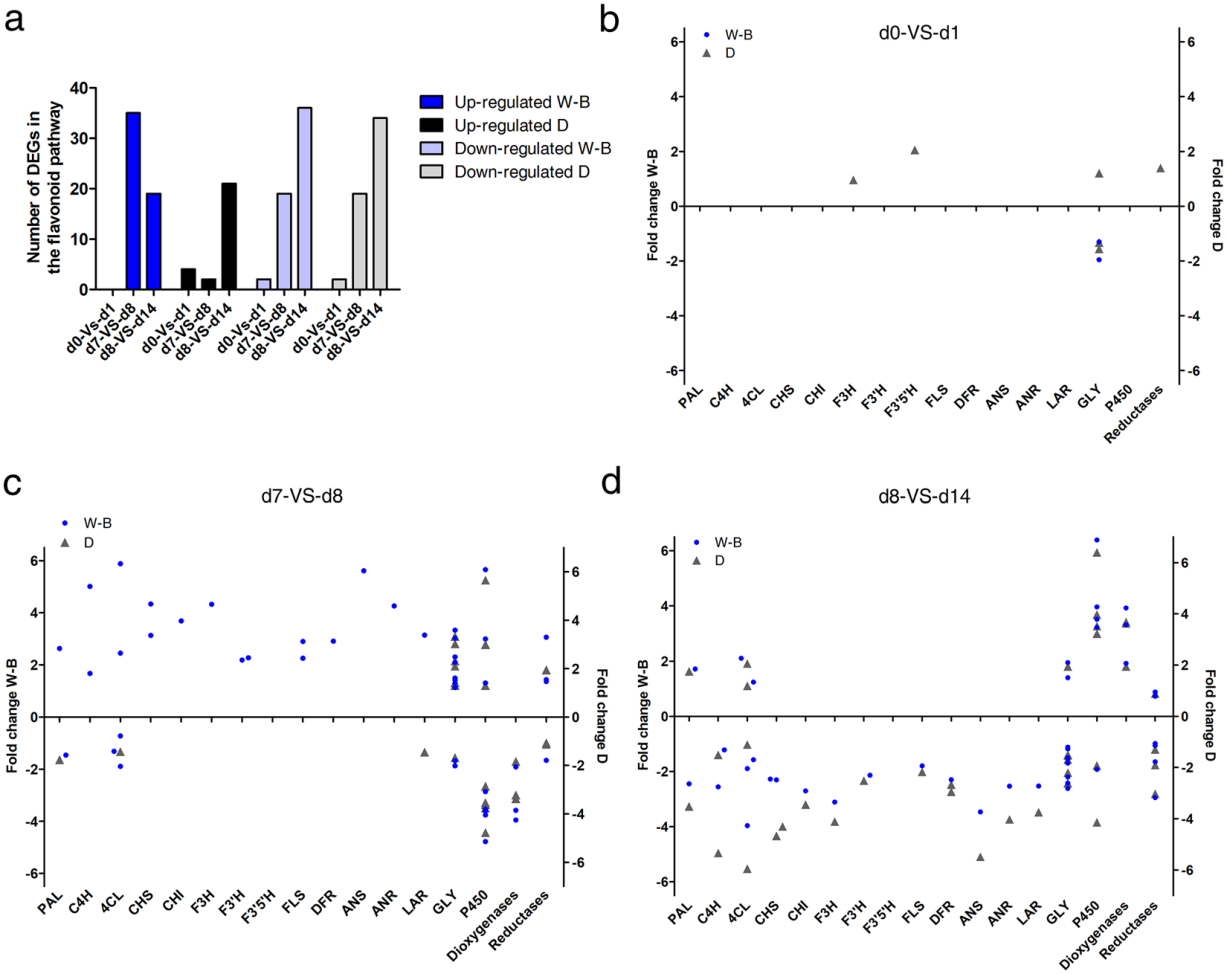
Diagrams of changes in expression levels of differentially expressed flavonoid genes. (**a**) Number of up and down regulated structural genes in the flavonoid pathway. (**b**–**d**) Fold changes for flavonoid genes at d0-VS-d1, d7-VS-d8 and d8-VS-d14. Blue dots for treatment W-B. Gray triangles for treatment D. Abbreviations: day (**d**), phenylalanine ammonia lyase (PAL); cinnamate 4-hydroxylase (C4H); 4-coumarate coenzyme A ligase (4CL); chalcone synthase (CHS); chalcone isomerase (CHI); flavanone 3-hydroxylase (F3H); flavanone 3′-hydroxylase (F3′H); Flavonoid-3′,5′-hydroxylase (F3′5′H); Flavonol synthase (FLS); dihydroflavonol-4-reductase (DFR); anthocyanidin synthase (ANS); anthocyanidin reductase (ANR); leucoanthocyanidin reductase (LAR); Glycosiltransferases (GLY); Cytochrome P450 enzymes (P450).

We further analyzed the DEGs using MapMan to classify individual gene responses in secondary metabolism and the flavonoid pathways. Interestingly, a higher variation in the number of genes of the flavonoid pathway were found for up regulated genes between the pairwise comparisons for both W-B and D compared with down-regulated genes (Fig. 3a). For W-B in the change from dark to white light at d0-VS-d1, two *UDP-glucosyltransferases* genes were down-regulated (Fig. 3b). Interestingly, after the change from white to blue light in d7-VS-d8 comparison, most of the flavonoid synthesis structural genes were up-regulated. In particular, late genes *ANR* and *ANS* had Log_2_ fold changes (LFC) of 4.2 and 5.6, respectively (Fig. 3c). During blue light treatment, comparison d8-VS-d14, the opposite pattern was found, with most of the structural genes reported as down-regulated, including the late genes *ANS*, *ANR* and *LAR* (LFC −3.4, −2.5 and −2.5 respectively) (Fig. 3d, Supplementary Table S3).

In the D condition at d0-VS-d1, the genes *F3H*, *F3′H*, *FLS* and *DFR* were up-regulated especially *F3′H* with 2.05 LFC (Fig. 3a,b). At d7-VS-d8, these same genes were down-regulated including an active copy of the *LAR* gene (LFC −1.4) (Fig. 3c). At the d8-VS-d14 most of the structural genes were down-regulated particularly late genes like *ANS*, *ANR* and *LAR* with LFC −5.48, −4.0, −3.7 respectively (Fig. 3d, Supplementary Table S3). Our results indicate that shift from white to blue light affected gene expression faster than permanent growth in the dark. The opposite trend in gene expression was observed when we compared d8 to d14 time points that could be resulting from negative feedback following long-term exposure to light. We further analyzed the top 30 up- and down- regulated DEGs at d8-VS-d14 for both treatments, searching putative genes linked to changes in the catechin profile. As a result, *ANS* and *CH4* were found strongly downregulated in the D treatment (Supplementary Table S4).

### Analysis of late genes of flavonoid biosynthesis using Co-expression networks

To identify genes co-regulated with flavonoid late genes directly producing catechins (flavan-3 ols), we performed co-expression network analysis using WGCNA^48^. A total of 16,634 and 16,526 genes for W-B and D respectively were used to perform the co-expression network. We generated 17 (W-B) and 19 (D) modules after creating the adjacency matrix (Supplementary Figs S6 and S7).

**Figure 4:**
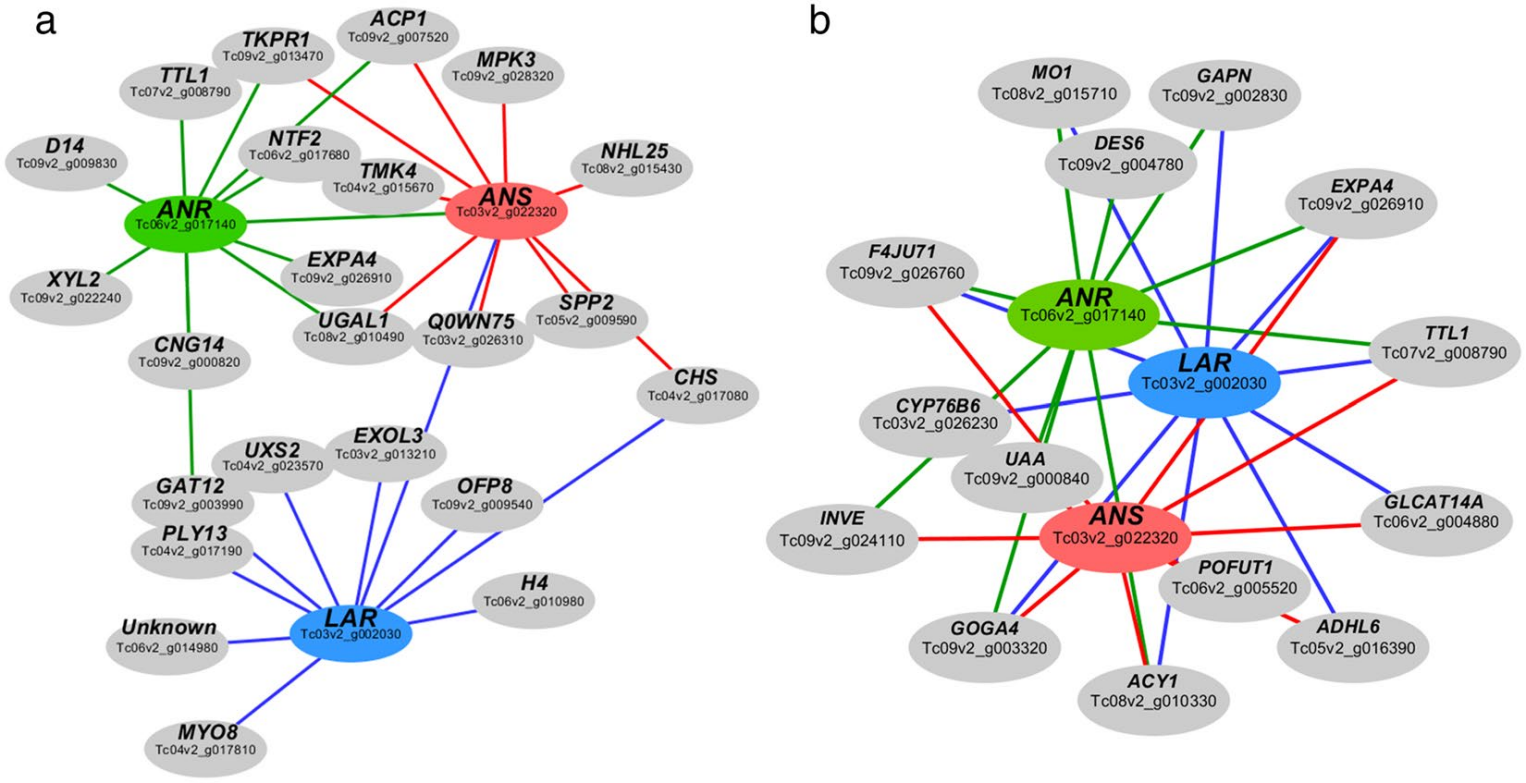
Co-expression network using late genes of flavonoid pathway *ANS*, *ANR* and *LAR* as seed nodes. (**a**) Subnetwork for W-B in the “Indianred4” module. (**b**) Subnetwork for D in the “Darkgreen” module. In both cases using top 30 of genes co-expressing with late biosynthetic genes.

For both treatments W-B and D, we selected the top 10 genes (i.e. those with the stronger pairwise correlations) co-expressing with each one of the late genes in flavonoid biosynthesis to be visualized with Cytoscape (Fig. 4). For the W-B network, late genes co-expressing with genes associated to different biological processes are detailed in Supplementary Table S5. Interestingly, *LAR* and *ANR* co-expressed with GAT12, a light responsive and *ANS* co-expressed with flavonoid pathway genes like *CHS* and *ANR*. Additionally, *ANS* co-expressed with both *LAR* and *ANR*, while no co-expression between *ANR* and *LAR* was detected (Fig. 4a). For D, the co-expression network is detailed in Supplementary Table S6. Interestingly, genes like *CYP76B6* (secondary metabolism), *INVE* (sucrose metabolism) and *DES6* (fatty acid metabolism) co-expressed with *LAR*, *ANS* and *ANR* respectively. In D network, more genes co-expressed simultaneously with all late genes indicating a common and shared processes in the dark, opposite to the light network, where late genes had more specialized partners (Fig. 4b).

### Analysis of differential expression of transcription factor genes

We next analyzed differential expression of genes encoding for transcription factors (TFs) involved in control of light induced responses as well as the onset of metabolism under stress. By using Plant Transcription Factor Database with our DEGs, we identified 578 non- redundant transcription factors (TFs) in our total set of DEGs for W-B and D conditions. For W-B, 83 TFs were common for d0-VS-d1, d7-VS-d8, and 264 overlapped for d7-VS-d8 and d8-VS-d14 timepoints where blue light responsive transcription factors are likely to be found. For D, 48 TFs were shared between d0-VS-d1 and d7-VS-d8, and 182 overlapped between d7-VS-d8 and d8-VS-d14. Interestingly, more transcription factors were expressed under W-B (532) than D (390) suggesting higher downstream activation under light treatments. Several families of TFs including MYB, bHLH, ERF, WRKY, and NAC were found overrepresented in the DEGs based on the normalized counts (Table 1, Supplementary Table S7).

**Table 1:** Overrepresented transcription factor families in W-B and D conditions.

Transcription factor family	Number of members
AGAMOUS-like	8
Auxin response factor	10
B3 family protein	10
bHLH family protein	45
C2H2 family protein	17
C3H family protein	14
ERF family protein	36
FAR1-related sequence	14
G2-like family protein	18
GATA transcription factor	8
LOB domain-containing protein	10
MYB family protein	53
NAC family protein	32
Nuclear factor Y	12
SCARECROW-like	9
Trihelix family protein	9
WRKY DNA-binding protein	34
Zinc Finger family (ZnF)	8

**Figure 5:**
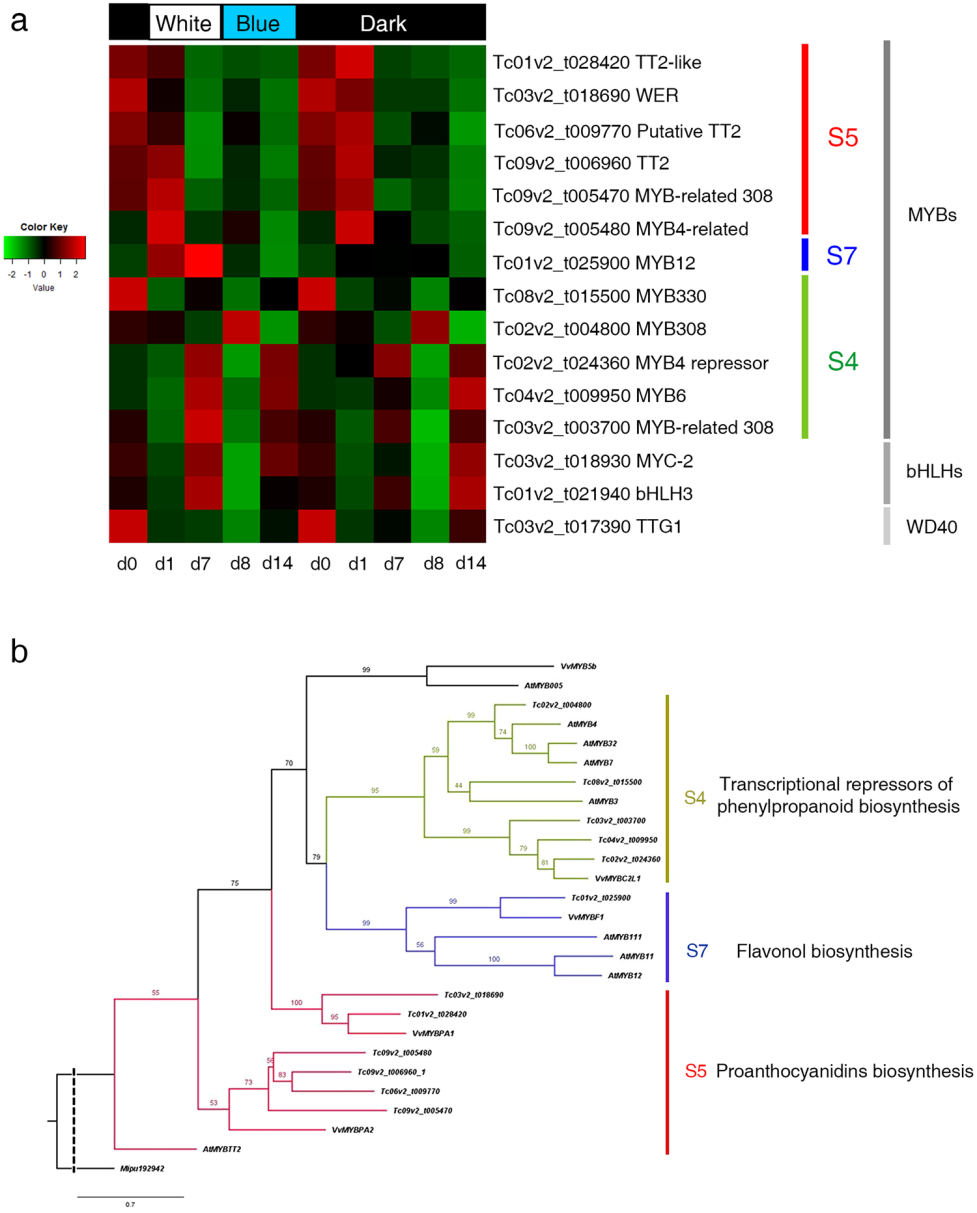
Expression patterns of MYB, bHLH and WD40 transcription factors and phylogeny of selected MYBs in cacao. (**a**) Heat map illustrating expression patterns of selected transcription factors of MBW complex at W-B and D treatment. (**b**) Phylogenetic analysis of selected MYBs genes associated with flavonoid pathway for cacao, Arabidopsis and grape. Abbreviations: day (**d**). Dotted line: shortened evolutionary distance.

To better assess which TFs could directly or indirectly regulate flavonoid accumulation, we focused in members of the MBW (MYB/bHLH/WD40) complex in all time points. In particular, our analyses targeted MYB genes because they have been linked to flavonoid biosynthesis in functional analyses in other plant species. To assess homology for the DEG MYB genes, we included all 53 differentially expressed MYB cacao genes in a phylogenetic analysis with all the MYBs reported for Arabidopsis (Supplementary Fig. S8). Then, we performed a heatmap and a second phylogenetic tree using 12 selected cacao MYB genes specifically involved in flavonoid biosynthesis (Fig. 5a). We classified them into 4 types following previous classifications^49^. Our results rescued those related with the subgroup 7 (S7; Flavonol biosynthesis), S5 (Proanthocyanidins biosynthesis) and S4 (Transcriptional repressors of phenylpropanoid biosynthesis). The group with most DEGs was S5, with six cacao genes up-regulated early in the W-B and D treatments at d0 and d1. One gene of the S7 group (Tc01v2_t025900, *MYB12*) was up-regulated at d1 and d7 only in W-B and six genes of the S4 group were up-regulated at day 7 and day 14 in both treatments. Finally, genes associated with *MYB5* were not found (Fig. 5b).

In order to assess homology for the copies in cacao, we performed a phylogenetic analysis using all the bHLH genes reported for Arabidopsis and the 45 bHLH differentially expressed for cacao (Supplementary Fig. S9). Among the bHLH genes involved in flavonoid biosynthesis *TT8*, *GL3* (*Glabra3)*, and *EGL3* (*Enhancer of Glabra3*) have been well characterized in Arabidopsis. We searched for their homologs in our transcriptomes, and we found that *AtGL3* and *AtEGL3* blasted to Tc03v2_t023390 in cacao. We found that this gene is expressed in all time points in both W-B and D, but did not show differential expression. Similarly, the homolog of *TT8*, Tc01v2_t023340, was not differentially expressed in our transcriptomes. Interestingly, in our phylogeny of bHLHs, two genes including the transcription factor *MYC2* (Tc03v2_t018930) and *bHLH3* (Tc01v2_t021940) were nested with *AtGL3* and *AtEGL3* and At*TT8* and both genes were upregulated at day 7 and day 14 for W-B and D, respectively. Finally, as for the WD40 homologs of the MBW complexes, we searched the homolog of Arabidopsis of *TRANSPARENT TESTA GLABRA 1*, *TTG1* in cacao (Tc03v2_t017390). This gene showed one peak of upregulation early for both treatments at day 1.

### Analysis of gene expression of light signal perception and signaling genes

**Figure 6:**
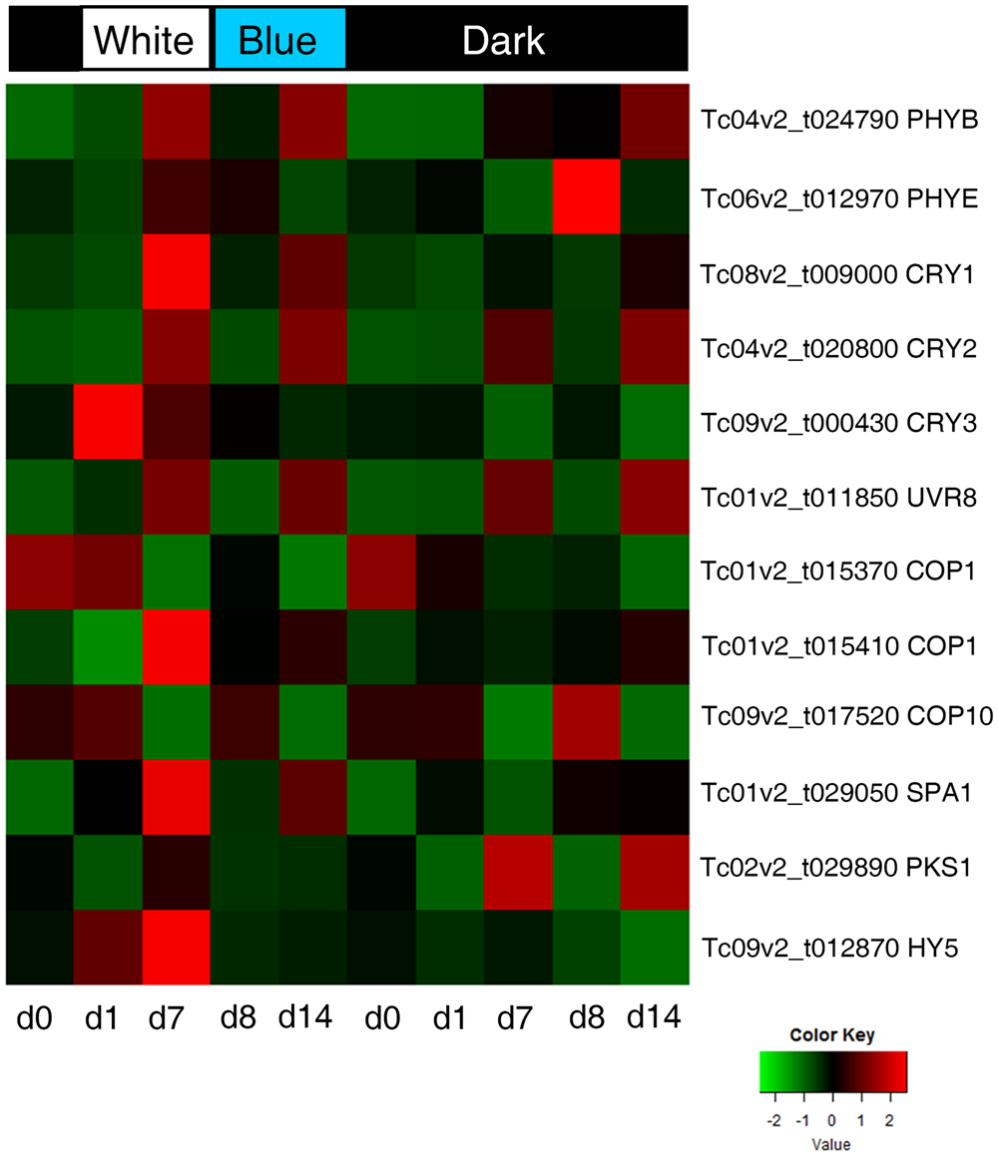
Heat map of selected light signaling genes over time in white-blue light versus dark. Treatments are summarized at the bottom. To the right are the codes assigned for each gene. Abbreviations: day (**d**).

Because light perception has been linked to flavonoid accumulation in plants^33,50^, we next explored regulation of light responsive genes in each time point using heat maps. From all DEGs we selected genes encoding for photoreceptors (i.e. phytochromes - *PHYs*, cryptochromes - *CRYs*, and *Uv-B Resistance 8* - *UVR8*) and downstream light signaling genes such as *Constitutive Photomorphogenesis 1 and 10* (*COP1, COP10*), *Suppressor Of PHYA* (*SPA1*), *Phytochrome Kinase Substrate 1* (*PKS1*) and *Long Hypocotyl 5* (*HY5*) (Fig. 6). In general, most of photoreceptors showed two peaks of upregulation, at d7 and d14, in W-B when compared to D. *CRY3* showed a strong up-regulation at d1 and d7 only in W-B. In D, *PHYB*, *PHYE* and *COP10* showed one peak up-regulation, particularly *PHYE*. Genes *CRY2*, *UVR8* and *PKS1* showed two peaks at d7 and d14. One of the copies of *COP1*, Tc01v2_t015370 was upregulated in both W-B and D at d0 and d1, instead the other copy, Tc01v2_t015410 was upregulated only in light at d7. Interestingly, *CRY1*, *COP1*, *SPA1* and *HY5* were upregulated only in W-B, in a manner opposite to *PKS1* been upregulated exclusively in D.

### qRT-PCR Validation of Differentially Expressed Genes

To validate further the RNA-seq results, we randomly selected 8 DEGs: *CHS*, *CHI*, *DFR*, *ANS*, *ANR*, *LAR*, *NAC* and *TT2-like* for qRT-PCR analysis 0, 1, 7, 8 and 14 days after treatment (Supplementary Table S8). The qRT-PCR showed the same patterns as the transcript abundance as determined by RNA-seq (Supplementary Fig. S10).

## Discussion

The proposed model for light-induced flavonoid biosynthesis includes a multi-level regulation by photoreceptors, negative regulators (i.e. COP1), light-response effectors (i.e. HY5), transcription factor complexes (MYB-bHLH-WD40, MBW), and structural genes directly promoting flavonoid biosynthesis (incl. *ANR*, *ANS*, *LAR*)^50^. The discussion will follow this direction in order to explain the progressive genetic bases resulting in an increased content of phenolic compounds in cacao cell suspensions in both W-B and D treatments along the time course of 14 days. To identify the genetic underpinnings of accumulation of (+)-catechin in W-B treatment we will also discuss co-expression networks constructed for major key structural late genes like *ANR*, *ANS* and *LAR*.

Our study demonstrated that *CRY1*, *COP1*, *SPA1* and *HY5* were strongly up-regulated only in W-B, in particular under white light at day 7. This is consistent with the known model for light perception and signaling, where the COP1/SPA complex interacts with photoreceptors like *CRY1*^51^ leading to a self-regulatory feedback loop by dissociation of *COP1*^33,52^. Low abundance of *COP1* in the nucleus allows *HY5*, the master positive regulator of light signaling, to accumulate and directly activate the flavonoid pathway, by binding to the promoter of *CHS*^33,53–55^. Our data suggested that the light signaling pathway that controls flavonoid biosynthesis is maintained in the cell cultures, as it occurs in plants^33^. In contrast, fewer genes, including *PHYE*, *COP10* and *PKS1*, were strongly up-regulated in D with *PKS1* overexpressed exclusively under this condition. The up-regulation of *PHYE* has also been detected under low fluence responses in promoting germination in Arabidopsis^56^. *PKS1* has been related to signal transduction of *PHYA* and *PHYB* in Arabidopsis^57^, however in cacao cell suspensions is only active in the dark, whereas in Arabidopsis has been shown to occur both in light and in dark and there are no additional reports on dark stimulated transcription for *PKS1*, which would point to a light independent role of *PKS1*, likely unlinked to *PHY* activation in cacao cell cultures.

As expected, major differences were observed between W-B and D regarding expression of photoreceptors. In this first line of genetic regulation immediate responses occur to specific wavelengths corroborating functional data gathered in Arabidopsis^58^. Additionally, our data provides evidence that production of phenolics also occurs in a light independent manner as demonstrated by the results from the cacao cultures incubated in the dark. This would suggest that photoreception is not essential for biosynthesis of phenolics *in vitro*. It is possible that blue light increases phenolic compounds as a defense mechanism as suggested by the early and strong response under the light change when compared to the dark.

Downstream of the light signaling genes we identified the MYB, bHLH and WD40 (MBW) transcription factors complex involved in the flavonoid pathway which binds to promoters of structural genes, including *ANR* and *LAR*^59–62^. The TT2–TT8–TTG1 complex controls the expression of the late biosynthetic genes promoting the accumulation of PAs^16,18^. In our data, several genes belonging to MYB and bHLH were found and the *TTG1* homolog in cacao (Tc03v2_t017390) with a WD40 domain was detected (Fig. 5). Three different expression trends were recorded for MYB genes. Those from the S5 group were expressed in a light independent manner at d0 and d1, whereas *MYB12*, was the only member of the S7 group expressed at d1 and d7 exclusively in the W-B treatment. S5 genes like *Tc-MYBPA* (Tc01v2_t028420) regulate PA synthesis by direct regulation of *LAR* and *ANR* both in grape and in cacao^26,63^, explaining polyphenol accumulation during both, W-B and D light regimes. Nevertheless, the difference of *MYB12* alone during the time course of the experiment in W-B when compared to D could result in earlier polyphenol production. Consistent with previously published reports from Arabidopsis, tobacco, and grape, our data suggests that the *MYB12* act as flavonol-specific activator induced only by the light treatment^29,64,65^. These results suggest that in *in vitro* cultures MYB proteins act as activators of the flavonoid synthesis pathway, similarly to their function *in planta* and that *MYB12* could be a candidate gene for manipulation of light induced flavonoid biosynthesis in cell suspension cultures.

The remaining *MYB* genes from group S4, well known repressors of the flavonoid pathway, include *AtMYB3*, *AtMYB7*, *AtMYB4*, and *AtMYB32* among others^66^. At d7 and d14 in both W-B and D treatments most repressors were up-regulated, immediately after the peaks of up-regulation for most of MYBs activators. A similar expression pattern has been recorded for *VvMYBC2L1* in grape berry development^67^. Interestingly, three cacao paralogs appear nested in the same subclade with *VvMYBC2L1*, suggesting cacao specific duplicates with repressive roles. Besides the function of S4 genes in negatively regulating the synthesis of phenolic compounds, they have been shown to fine tune flavonoid levels in grape^68^. Altogether, the fine tuning of *MYB* activators and repressors in the time course, in addition to the exclusive upregulation of *MYB12* in the light, could explain the catechin profile differences among treatments.

As for the bHLH genes, the expression pattern of *Tc-MYC-2* and *Tc-bHLH3* was similar to that of S4 MYBs repressors, however a crosstalk between bHLH and MYB repressors has not been reported. The bHLH homologs found here are involved in the positive regulation of flavonoids in other plant species^69–71^. *MYC2* positively regulates flavonoid biosynthesis in response to jasmonate (JA) in Arabidopsis^72^. The targeted search for *Jasmonic acid-amido synthetase* in our analyses revealed its upregulation at d8-VS-d14, which could explain the downstream flavonoid accumulation by *TcMYC-2*. In addition, *MYC2* has been linked to anthocyanin synthesis^69^, but in cacao cell suspensions, *Tc-MYC-2* seems to also regulate PA synthesis. The expression of *Tc*-*bHLH3*, which is turned on at d7 in W-B and at d14 in D, reveals that in cacao cell cultures this gene is active in a light independent manner. The interaction between bHLH3 and MYB12 is known to promote PA synthesis in red-fleshed apple^73^, thus, as *Tc-bHLH3* and *Tc-MYB12* only overlap in W-B at d7 their interaction could only explain PAs synthesis in the light. However, PAs are also synthesized in D, suggesting that in the dark *Tc*-*bHLH3* has alternative partners. Finally, the cacao homolog in the WD-40 clade, named *TTG1* presented a co-regulation with *TcMYB330* (a MYB repressor, the ortholog of *AtMYB3*) with upregulation at d1 for both treatments. No reports are available for interactions between TTG1 and MYB3 homologs, except for the interaction between the MYB homolog repressor of anthocyanins *CAPRICE* (*CPC*) and *TTG1* in Arabidopsis^74,75^. Based on our co-expression data, we hypothesize that in cacao cell cultures *Tc-TTG1* and *Tc-MYB330* are likely interacting, but their contribution to flavonoid biosynthesis is still unclear and further functional analysis should be done.

Downstream of the TFs are the structural genes of the flavonoid pathway. Two key structural enzymes unique to PA biosynthesis and which determine the catechin profiles are *anthocyanidin reductase* (*ANR*) and *leucoanthocyanidin reductase* (*LAR*)^76^. Differences in concentration of (+)-catechin between W-B and D could be attributed to differences in expression of *ANR* or, more likely, *LAR* (Fig. 3). *ANR* in tea participates in the production of both epicatechin and catechin using cyanidin as substrate^77^. Thus, it is possible that the same role is maintained in cacao cell cultures. More interesting is the differential regulation of *LAR*, as this enzyme catalyzes the synthesis of catechin (2,3-*trans*-flavan-3-ol) from 3,4-cis-leucocyanidin^55^. *TcLAR* was upregulated three-fold at d7-VS-d8 in W-B and one-fold in D. Overexpression of *TcLAR* in tobacco produced more epicatechin than catechin^25^, the opposite of what is seen in cacao cultures where more *TcLAR* results in a 2-fold increase of catechin in W-B. This suggests differences between the heterologous transformation *versus* the endogenous activation of *TcLAR* in cacao cell cultures. Similarly, when *TcLAR* is overexpressed in *ldox (ans)* Arabidopsis mutants, which are impaired in anthocyanins and PAs synthesis, there is a significant increase of catechin, but also a slight increase of epicatechin^25^. The same dual functionality of *LAR* was recently demonstrated in *Medicago*, where LAR converts 4β-(S-cysteinyl)- epicatechin back to epicatechin, the starter unit in PAs polymerization^78^. *TcLAR* has also been evaluated *in vitro* where it lacks a dual enzymatic activity as shown by the exclusive catechin synthesis^25^. Our data is consistent with the *in vitro* data, as catechin synthesis preferentially occurred at d7 vs d8 at WB during *TcLAR* overexpression. Altogether, the available data points to differential roles of TcLAR *in vivo* and *in vitro*, postulating this enzyme as a key target for metabolic engineering able to modify catechin profiles in cell suspensions.

Functional differences of *TcLAR* in W-B versus D could also be explained by changes in the interacting partners (Fig. 4). Our co-expression analyses revealed that in W-B, *TcLAR* had a strong interaction with *GAT12* (*GATA transcription factor 12*), also present in the top 30 differentially expressed genes. GATA mediates the crosstalk between brassinosteroids (BR) and light-signaling pathways^79^. Additionally, the relation between the BR and the flavonoid pathway has been reported^80^. Thus, it is possible that BR modulates catechin production in cacao cell cultures. The metabolism in the D treatment is far less specialized, as both *ANS* and *LAR* interact with *CYP76B6* (Putative Geraniol 8-hydroxylase), a cytochrome P450 monooxygenase involved in the biosynthesis of iridoid monoterpenoids^81^ which participate in general signaling of secondary metabolites in cell cultures. The co-expression analysis comparison results in a change from shared partners among *ANS*, *ANR* and *LAR* in the dark to a specialized network for each enzyme in W-B, suggesting different functional capabilities for these enzymes under light *in vitro*.

The cross-talk between light regulated genes, transcription factors and flavonoids is one of the most promising for increased flavonoid production in crops and likely improved food quality^27,50^. The existence of light regulatory units (LRUs) in the promoter region of structural genes like *CHS*, *F3H* and *FLS*^28,82^ suggest their direct regulation by light, however for late genes including *ANS*, *ANR* and *LAR* it remains unknown. Here we show that exposure of cacao cell cultures to different light treatments results in catechin profile changes and provide a platform for dissecting complex genetic networks. Cell cultures allowed the synchronizing of the light signaling responses due to their homogeneity. We determined that light can effectively regulate flavonoid profiles as it changes catechin to epicatechin ratios and we postulated candidate genes (*MYB12*, *HY5*, *ANR* and *LAR*) as potential targets of genetic engineering to manipulate the production of cacao polyphenols. Co-expression networks further revealed that light promotes specialization of interacting partners for late biosynthetic genes. Further functional analysis will provide a deeper understanding of the regulatory network of the biosynthesis of catechins. The *in vitro* cacao cell suspension system and the DEGs identified in this study will enable the development of new biotechnological tools for the generation of value-added plants with optimized flavonoid content.

## Materials and Methods

### Plant Material and growth conditions

Mature, eight-month-old cacao pods of the Trinitario ecotype (coded as BIOA) were collected in the commercial crops of Compañía Nacional de Chocolates in the region of San Vicente de Chucurí- Santander, Colombia (06° 53′ 00′′N; 73° 24′ 50′′ W). Cacao cell suspensions were induced following Gallego *et al*.^83^. The cultures were maintained at 23 ± 2 °C under cool-white fluorescent lamps (12 µmol m^−2^. s ^−1^) for 16 hours light at 100 rpm. After 12 days, cell suspensions were filtered using a 300 µm mesh and an inoculum (4 g/L dry weight) was used for establishing cultures. These were sub-cultured every 7 days under dark conditions in order to prepare the suspensions for light treatments (see below). Media composition was prepared according to Gallego *et al*.^83^

### Light treatments

Cell suspension cultures were exposed to two light treatments during a time course of 14 days using light-emitting diode (LED) lights. In the first treatment, we evaluated a sequential exposure to white LED followed by exposure to blue LED 7 days each (W-B). In the second treatment, cells were kept in the dark for 14 days (D). In both treatments, the day 0 was considered as the reference point for flavonoid production and fresh media was added on day 7. White and blue LED arrays were designed to give an intensity of 60 µmol m^−2^ s^−1^ under a photoperiod of 16 hours light and 8 hours dark. Erlenmeyer flasks of 100 mL containing 30 mL of cells were sampled at days 0, 1, 7, 8 and 14 during the time course. Four replicates were collected for each time point and treatment (W-B and D). Cell suspension samples were preserved in RNA Later® (ratio 1:5) and stored at −20 °C.

### Quantification of total polyphenols, catechins and proanthocyanidins (PAs)

Extraction and quantification of total polyphenol content (TPC) were performed following Londoño *et al*.^84^ on days 0, 7, and 14 during the time course. Dried powdered samples (50–100 mg) were extracted with a mixture of water/isopropyl alcohol mix (40:60) and sonicated for 1 hour with A~96 = 230 watts (Misonix, S-4000, USA). Samples were centrifuged at 10,000 rpm for 5 minutes at 4 °C. The supernatant was transferred and taken to known volume with distilled water. Folin-Ciocalteau method was used to quantify according to Londoño *et al*.^84^. Polyphenol extracts were injected in a High-Performance Liquid Chromatographer (HPLC, Agilent Technologies, series 1260), equipped with quaternary pump, automatized injector, fluorescent detector (FLD) and diode-array detector (DAD). A total of 200 µl of each extract were diluted to 2 ml using acetic acid solution 0.1%. Chromatographic resolution of analytes in the extracts were made through a Zorbax Eclipse XD-C18 column (4.6 × 250 mm, 5 µm particle size) with constant flow of 1 m/min. Mobile phases were acetic acid 0.1% as A solvent and methanol as B solvent, starting in 10% B for 6 min, after 20% for 14 min, up again to 22.5% for 3 min, down 20% for 8 min and lastly returned to the initial conditions for 6 min. Total running time was 37 min. Catechins ((+)- catechin and (−)- epicatechin)) were measured using HPLC and were monitored in the fluorescent detector at PMT 10 (photomultiplicator) with excitation and emission wavelengths of 280 and 315 nm respectively. The average values of three independently sampled suspensions were calculated. Extraction and quantification of proanthocyanidins were according Liu *et al*.^25^. In short, dried 50 mg of suspensions were used to extract consecutively soluble and insoluble PAs with acetone:water:acetic acid (70%:29.5%:0.5%) and buthanol:HCl (95%:5%) respectively. The colorimetric p-dimethylamino-cinnamaldehyde (DMACA) method was used for quantification of soluble PAs. Absorption was measured at 640 nm and procyanidin B2 was used as standard for quantification. For insoluble PAs, absorbance was measured at 550 nm and PAs were calculated as cyaniding equivalents using cyanidin 3 glucoside. Soluble and insoluble PAs were added to give the total PAs content.

### RNA extraction, library preparation and sequencing

Four replicates were sampled at each time point (d0, d1, d7, d8 and d14) from the W-B and D treatments for total of 36 samples. Total RNA was extracted from each cell suspension taking 0.1 mL approximately of pellet volume using combined protocols of PureLink Plant RNA Reagent (Ref: 12322012, Thermo Fisher Scientific) and RNeasy Plus Universal Mini Kit (Cat No. 73404, QIAGEN) following the manufacturer’s protocol. RNA concentrations and quality were measured using an Agilent 2100 bioanalyzer and a minimal RIN number of 7 was used for sequencing. The construction of the cDNA libraries and RNAseq were performed by the Genomics Core Facility at Penn State University (University Park, USA). cDNA libraries were prepared with a TruSeq stranded mRNA library prep Kit (cat# RS-122-2101, Illumina, San Diego, CA, USA). The libraries were sequenced on a HiSeq™ 2500 (Illumina) using single-end runs of 100 nt.

### Mapping and functional annotation

Quality parameters including GC-content and phred scores were applied to filter the reads. After filtrate low quality raw reads, mapping to the Criollo Cacao Genome V2^85^ was performed by using HISAT^86^. The sequences were annotated using Blast2Go (e-value <10^−5^) and the Cacao genome as a reference^87^. The following public protein databases were used for the enrichment analyses: Clusters of Orthologous Groups (COGs)^88^, Kyoto Encyclopedia of Genes and Genomes (KEGG)^89^, and Gene Ontology (GO) protein database^90^. Gene expression levels were normalized and differential expression analyses were conducted using Deseq2^91,92^. Three pairwise comparisons were performed within each W-B and D condition to identify differentially expressed genes (DEGs) at different time points. Comparisons were made between 0 and 1 day (d0-VS-d1), 7 and 8 days (d7-VS-d8) and 8 and 14 days (d8-VS-d14). DEGs were identified when the False Discovery Rate (FDR) was ≤0.05 and p-value was <0.05. These putative DEGs were similarly subjected to Gene Ontology (GO) enrichment analysis and KEGG Pathway enrichment analysis to investigate functions and pathways affected over the time course. GO and KEGG enrichment analyses were performed using KOBAS software^93^ to test the statistical enrichment of terms associated with DEGs. Mapman^94^ was used to complement the analysis of pathway regulation from KEGG. A FDR <0.05 was used as the threshold to determine significant GO/KEGG enrichment of the gene sets. Additionally, we used the Plant Transcription Factor Database^95^ to annotate DEGs in all the pairwise comparisons with the best transcription factor hits in *Arabidopsis thaliana*.

### Phylogenetic analyses of differentially expressed transcription factors genes

Three different phylogenetic analyses were performed. The first two included differentially expressed transcription factors belonging to the MYB (53) and bHLH (45) gene families with the reported homologs present in the *Arabidopsis thaliana* genome^19,96^ in order to identify orthologous genes. Full coding sequences from 144 *bHLH* genes and 125 *MYB* from Arabidopsis were retrieved from Phytozome^97^. A third analysis was done including twelve cacao MYB genes involved in the flavonoid biosynthesis pathway in a larger matrix consisting of 9 *MYB* genes from Arabidopsis and 5 MYBs from grape (including VvMYBC2-L1^68^, VvMYBPA1^63^, VvMYBPA2^98^, VvMYBF1^29^ and VvMYB5b^99^) also retrieved from Phytozome. Full coding sequences of transcription factors (TFs) aligned with the ClustalW algorithm were used to construct the phylogenetic trees using RAxML^100^ in CIPRES Gateway platform^101^. Up to 1000 Bootstrap replicates were run to assess support for the clades found.

### Correlation analysis

A weighted gene co-expression network was constructed using WGCNA R package^48^ with RNA-seq count data. All expressed genes under W-B and D conditions were normalized and genes with low counts (raw counts) lower than 50 were removed. Networks were visualized using Cytoscape V3.5.1^102^. To simplify the display of the network and to focus on relevant relationships, only edges of the corresponding TOM similarity measure above a threshold of 0.23 are shown. Special attention was given to the top 10 of genes associated with each *ANS*, *ANR* and *LAR*, the late structural genes of the flavonoid pathway.

### Quantitative PCR

Real-time quantitative reverse transcription-PCR (qRT-PCR) was used to validate gene expression patterns identified by the RNAseq analysis. Aliquots from the RNA used for RNAseq experiments were used as template for cDNA synthesis. RNA was treated with RNase-free DNase (Promega, Cat. M6101) following the manufacturer’s protocol. A total of 0.3 µg of treated RNA were reverse transcribed by MMuLV Reverse Transcriptase (New England Biolabs, Ipswich, MA, USA) using oligo-(dT)15 primers. Primers were designed for *CHS*, *CHI*, *DFR*, *ANS*, *ANR*, *LAR*, *TT2-like* and *NAC* based on the sequences available for the Criollo genome database. *ACTIN-7* and *UBIQUITIN* were selected as endogenous controls. qRT-PCR was performed in a total reaction volume of 10 μL containing 4 μL of diluted cDNA (1:8), 5 μL of SYBR Premix, 0.2 μL of Rox (TaKaRa, Mountain View, CA, USA), and 0.4 μL of each 5 μM primer. Four technical replicates were made for each sample. qRT-PCR was conducted in a real time PCR System (Applied Biosystem Step One Plus, Nutley, NJ, USA).

### Statistical analysis

Statistical significance of gene expression between pairwise comparisons was determined analyzing integral readcounts per gene with DESeq2^92^. ANOVA and TuckeyHSD test were used in the statistical analysis of phenolic quantifications using R software.

## Electronic supplementary material

Supplementary Figures

Supplementary tables

## Data Availability

The RNA-Seq data used in this research is available in NCBI with GEO/AdrianaGallego.
