# Supplementary material for: Transcriptomic analyses of cacao cell suspensions in light and dark provide target genes for controlled flavonoid production
Source: Sci Rep. 2018 Sep 11;8:13575. doi: 10.1038/s41598-018-31965-7 (PMC6134037; doi:10.1038/s41598-018-31965-7)
Supplement: Supplementary file 1 — Supplementary Figures [file 41598_2018_31965_MOESM1_ESM.pdf]

Transcriptomic analyses of cacao cell suspensions in light and dark provide target genes for controlled flavonoid production

Adriana M. Gallego[1], Luisa F. Rojas[2], Oriana Parra[1], Héctor A. Rodriguez[3], Juan C. Mazo Rivas[4], Aura Inés Urrea[1], Lucía Atehortúa [1], Andrew S. Fister[5], Mark J. Guiltinan[5], Siela N. Maximova[5*] and Natalia Pabón-Mora[6*]

1. Universidad de Antioquia, Grupo de Biotecnología, Medellín, Colombia
2. Universidad de Antioquia, Grupo de Biotecnología-Escuela de Microbiología, Medellín, Colombia
3. Corporación para Investigaciones Biológicas and Departamento de Ciencias Agronómicas, Facultad de Ciencias Agrarias, Universidad Nacional de Colombia, UNALMED-CIB, Medellín, Colombia
4. Compañía Nacional de Chocolates, Medellín, Colombia
5. Department of Plant Science, Pennsylvania State University, University Park, PA, United States
6. Universidad de Antioquia, Instituto de Biología, Grupo Evo-Devo en Plantas, Medellín, Colombia

*Corresponding authors. e-mail: snm104@psu.edu, lucia.pabon@udea.edu.co

Figure legends:

Figure S1. Total proanthocyanidins (PAs) content under light treatments. Error bars represent the standard deviation.

Figure S2. Histogram of gene ontology classification for all mapped genes

Figure S3. Histogram of genes COG classification for all mapped genes

Figure S4. Functional categories of DGEs based on Gene Ontology (GO) for W-B condition. Significantly enriched GO categories (P-value < 0.05) were analyzed in pairwise comparisons (0d-VS-1d, 7d-VS-8d, and 8d-VS-14d). The results are summarized as percentage of genes in three main categories: cellular component, molecular function, and biological process.

Figure S5. Functional categories of DGEs based on Gene Ontology (GO) for Dark condition. Significantly enriched GO categories (P-value < 0.05) were analyzed in pairwise comparisons (0d-VS-1d, 7d-VS-8d, and 8d-VS-14d). The results are summarized as percentage of genes in three main categories: cellular component, molecular function, and biological process.

Figure S6. Coexpression network analysis of W-B condition in cell suspensions transcriptomes using WGCNA. Dendrogram and modules for 16,634 genes clustered in 17 modules shown in different colors after merging process with height cut of 0.3, corresponding to correlation of 0.7 to merge.

Figure S7. Coexpression network analysis of D condition in cell suspensions transcriptomes using WGCNA. Dendrogram and modules for 16,526 genes clustered in 19 modules shown in different colors after merging process with height cut of 0.3, corresponding to correlation of 0.7 to merge.

Figure S8. Schematic representation of the relationships between the different R2R3-MYB subgroups for Cacao and Arabidopsis. Analysis inferred using RAxML with up to 1000 bootstraps with CDS full length MYB sequences. The subgroups were designated as previously reported[1]. MYB Mipu192942 from *Micromonas Pusilla* was used as outgroup.

Figure S9. Schematic representation of the relationships between the different bHLH genes for Cacao and Arabidopsis. Analysis inferred using RAxML with up to 1000 bootstraps and CDS full length bHLH sequences. The subgroups were designated as previously reported[2]. The bHLH Mipu55764 from *Micromonas Pusilla* was used as outgroup. Cluster with ATTT8, AtEGL3 and AtGL1 is highlighted in pink.

Figure S10. qRT-PCR analysis of differentially expressed genes in cacao cell suspensions. Transcript levels and qRT-PCR results of 8 randomly selected genes from RNA-sequencing. The left y-axis shows the relative gene expression levels analyzed by qPCR (gray columns). The right y-axis indicates the corresponding expression data of RNA-seq (black dots). The x-axis represents the time (days) of light/dark exposure. Bars represent SD (n = 3).

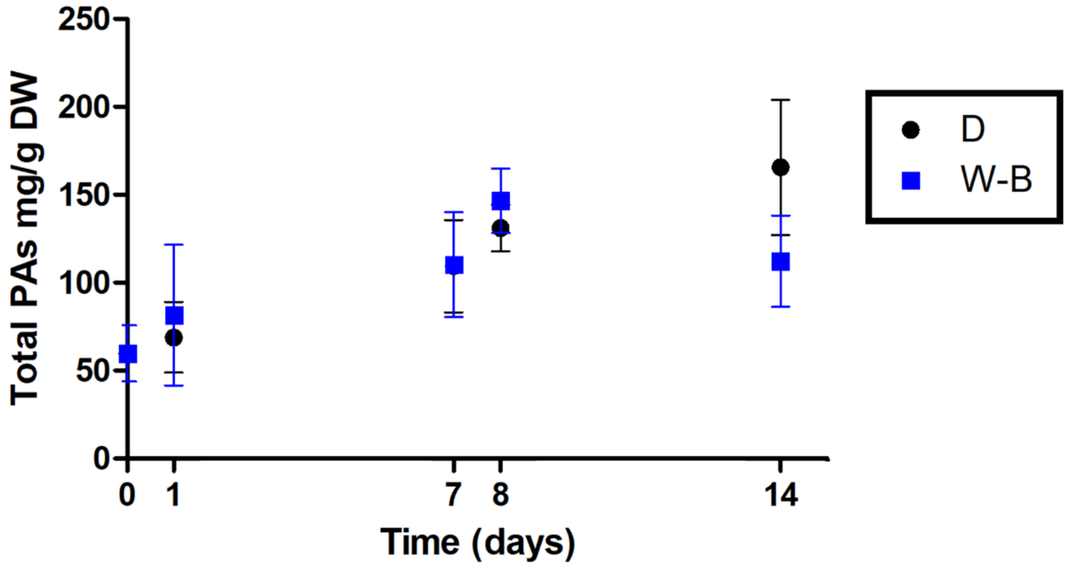

Figure S1. Total proanthocyanidins (PAs) content under light treatments. Error bars represent the standard deviation.

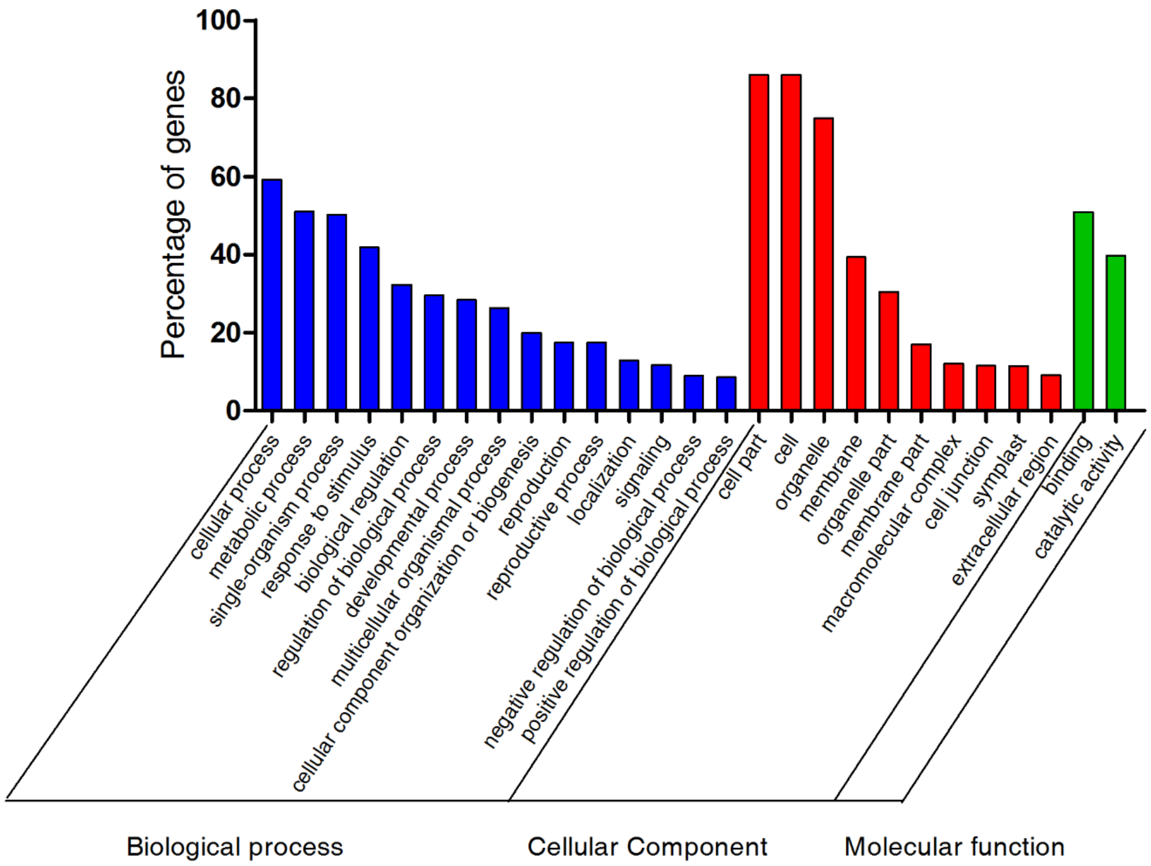

Figure S2. Histogram of gene ontology classification for all mapped genes.

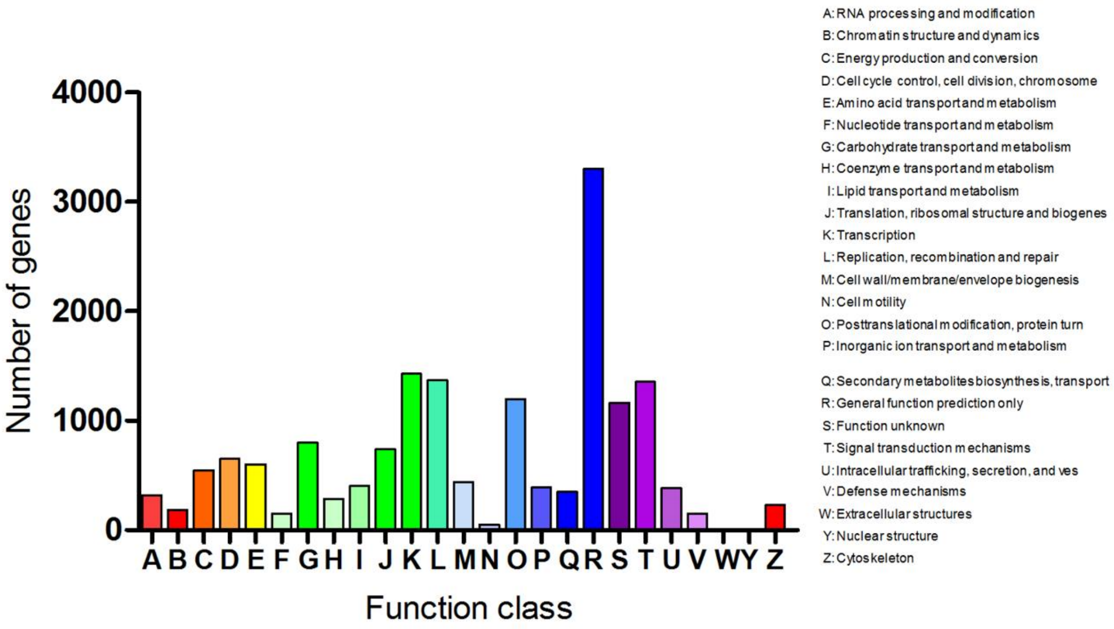

Figure S3. Histogram of genes COG classification for all mapped genes

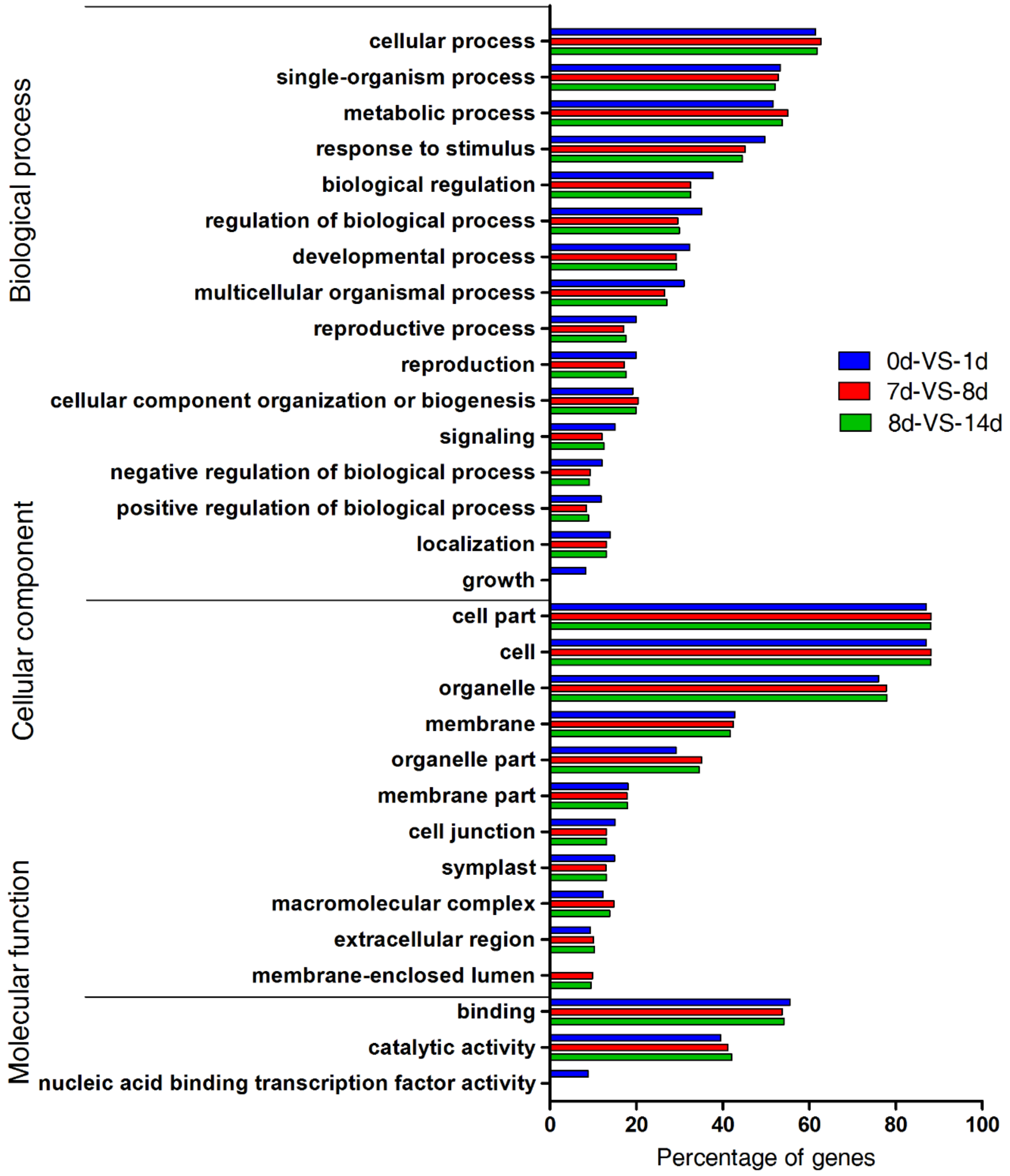

Figure S4. Functional categories of DGEs based on Gene Ontology (GO) for W-B condition. Significantly enriched GO categories (P-value < 0.05) were analyzed in pairwise comparisons (0d-VS-1d, 7d-VS-8d, and 8d-VS-14d). The results are summarized as percentage of genes in three main categories: cellular component, molecular function, and biological process.

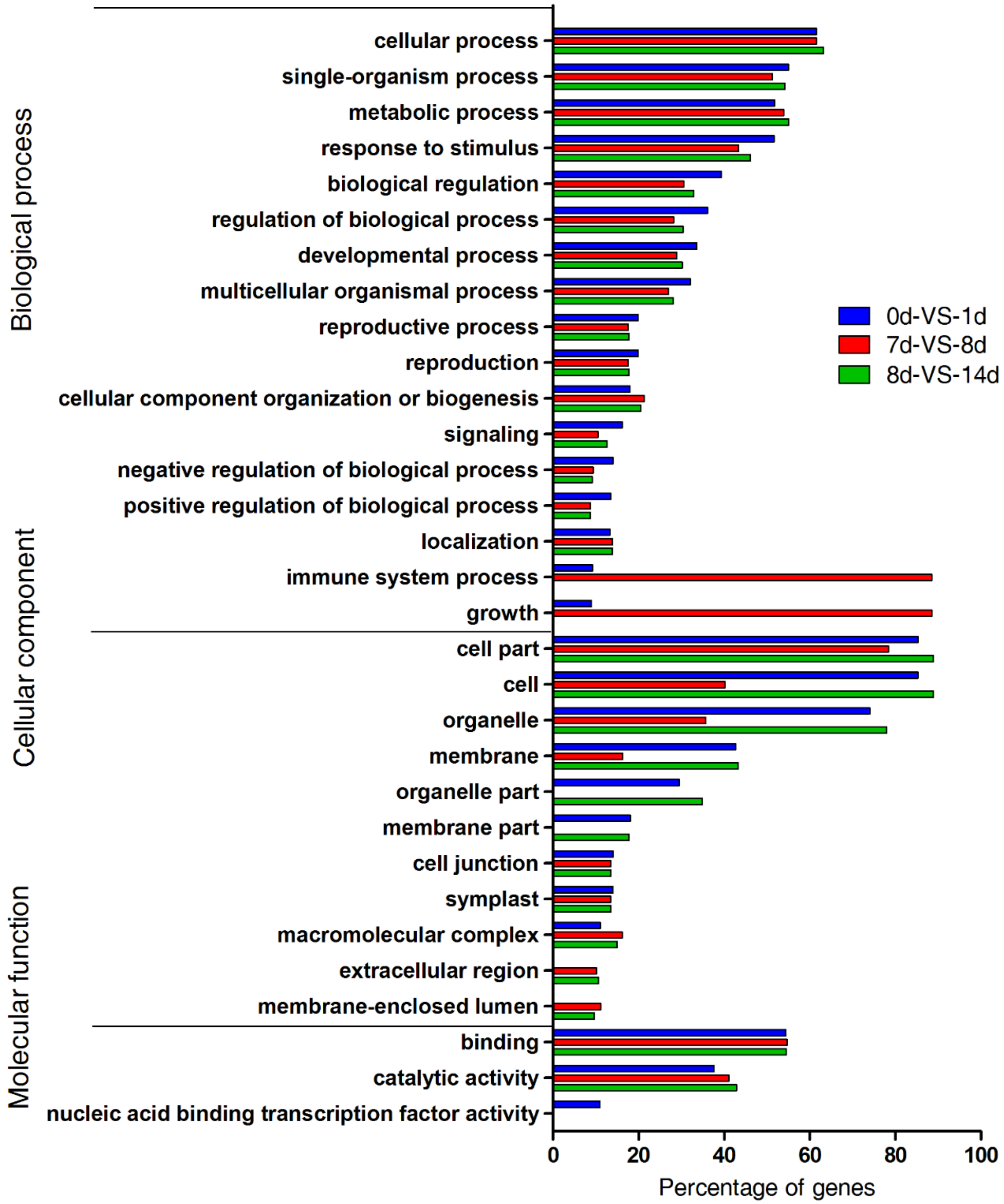

Figure S5. Functional categories of DGEs based on Gene Ontology (GO) for Dark condition. Significantly enriched GO categories (P-value < 0.05) were analyzed in pairwise comparisons (0d-VS-1d, 7d-VS-8d, and 8d-VS-14d). The results are summarized as percentage of genes in three main categories: cellular component, molecular function, and biological process.

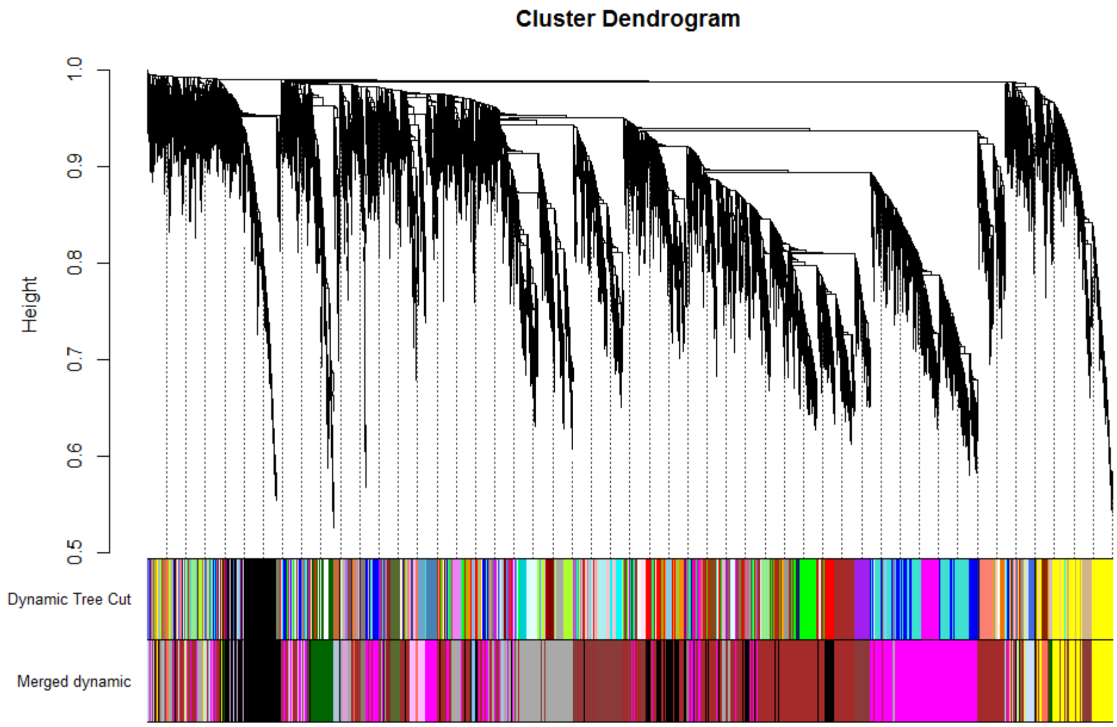

Figure S6. Coexpression network analysis of W-B condition in cell suspensions transcriptomes using WGCNA. Dendrogram and modules for 16,634 genes clustered in 17 modules shown in different colors after merging process with height cut of 0.3, corresponding to correlation of 0.7 to merge.

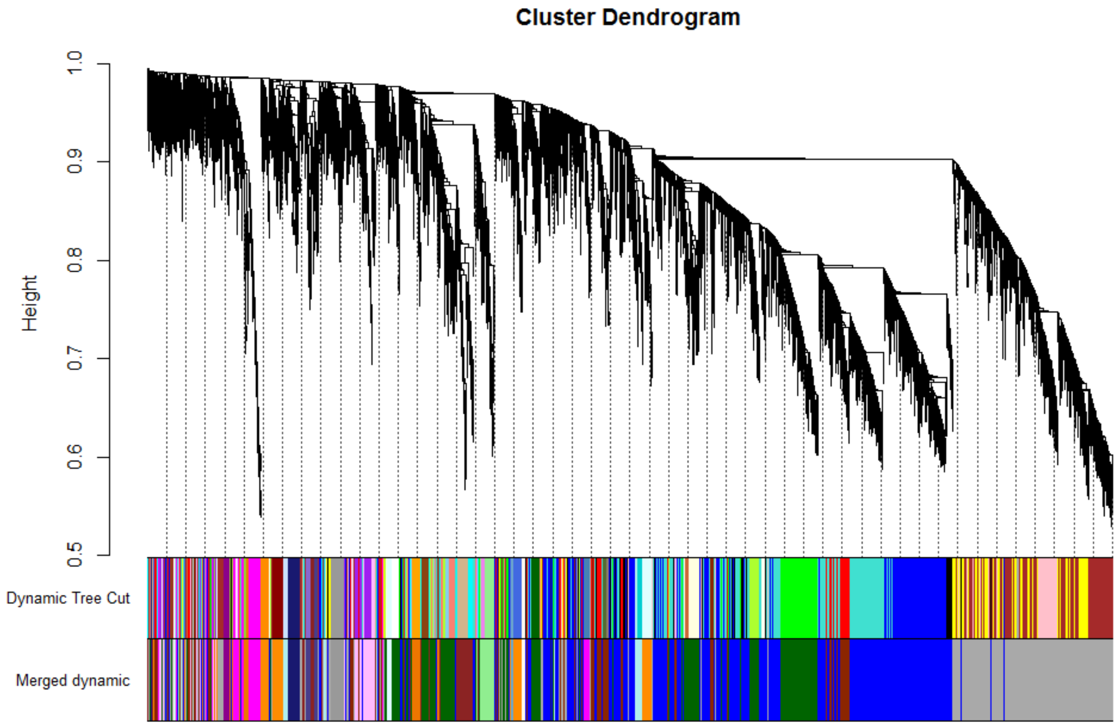

Figure S7. Coexpression network analysis of D condition in cell suspensions transcriptomes using WGCNA. Dendrogram and modules for 16,526 genes clustered in 19 modules shown in different colors after merging process with height cut of 0.3, corresponding to correlation of 0.7 to merge.

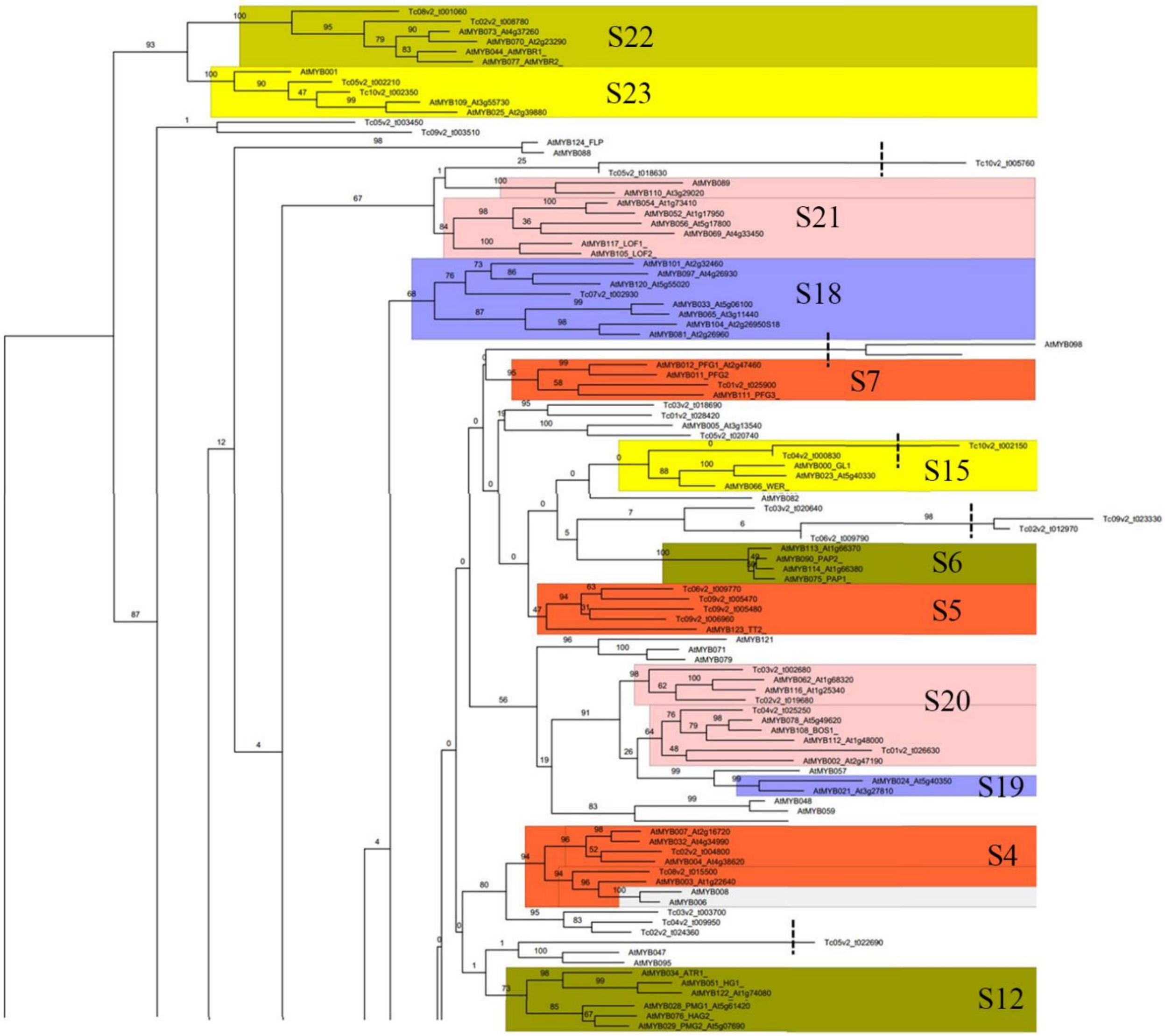
S22
S23
S21
S18
S7
S15
S6
S5
S20
S19
S4
S12
Tc08v2_t001060
Tc02v2_t008780
AtMYB073_At4g37260
AtMYB070_At2g23290
AtMYB044_AtMYBR1_
AtMYB077_AtMYBR2_
AtMYB001
Tc05v2_t002210
Tc10v2_t002350
AtMYB109_At3g55730
AtMYB025_At2g39880
Tc05v2_t003450
Tc09v2_t003510
AtMYB124_FLP
AtMYB088
Tc05v2_t018630
Tc10v2_t005760
AtMYB089
AtMYB110_At3g29020
AtMYB054_At1g73410
AtMYB052_At1g17950
AtMYB056_At5g17800
AtMYB069_At4g33450
AtMYB117_LOF1_
AtMYB105_LOF2_
AtMYB101_At2g32460
AtMYB097_At4g26930
AtMYB120_At5g55020
Tc07v2_t002930
AtMYB033_At5g06100
AtMYB065_At3g11440
AtMYB104_At2g26950S18
AtMYB081_At2g26960
AtMYB098
AtMYB012_PFG1_At2g47460
AtMYB011_PFG2
Tc01v2_t025900
AtMYB111_PFG3_
Tc03v2_t018690
Tc01v2_t028420
AtMYB005_At3g13540
Tc05v2_t020740
Tc10v2_t002150
Tc04v2_t000830
AtMYB000_GL1
AtMYB023_At5g40330
AtMYB066_WER_
AtMYB082
Tc03v2_t020640
Tc09v2_t023330
Tc02v2_t012970
Tc06v2_t009790
AtMYB113_At1g66370
AtMYB090_PAP2_
AtMYB114_At1g66380
AtMYB075_PAP1_
Tc06v2_t009770
Tc09v2_t005470
Tc09v2_t005480
Tc09v2_t006960
AtMYB123_TT2_
AtMYB121
AtMYB071
AtMYB079
Tc03v2_t002680
AtMYB062_At1g68320
AtMYB116_At1g25340
Tc02v2_t019680
Tc04v2_t025250
AtMYB078_At5g49620
AtMYB108_BOS1_
AtMYB112_At1g48000
Tc01v2_t026630
AtMYB002_At2g47190
AtMYB057
AtMYB024_At5g40350
AtMYB021_At3g27810
AtMYB048
AtMYB059
AtMYB007_At2g16720
AtMYB032_At4g34990
Tc02v2_t004800
AtMYB004_At4g38620
Tc08v2_t015500
AtMYB003_At1g22640
AtMYB008
AtMYB006
Tc03v2_t003700
Tc04v2_t009950
Tc02v2_t024360
Tc05v2_t022690
AtMYB047
AtMYB095
AtMYB034_ATR1_
AtMYB051_HG1_
AtMYB122_At1g74080
AtMYB028_PMG1_At5g61420
AtMYB076_HAG2_
AtMYB029_PMG2_At5g07690

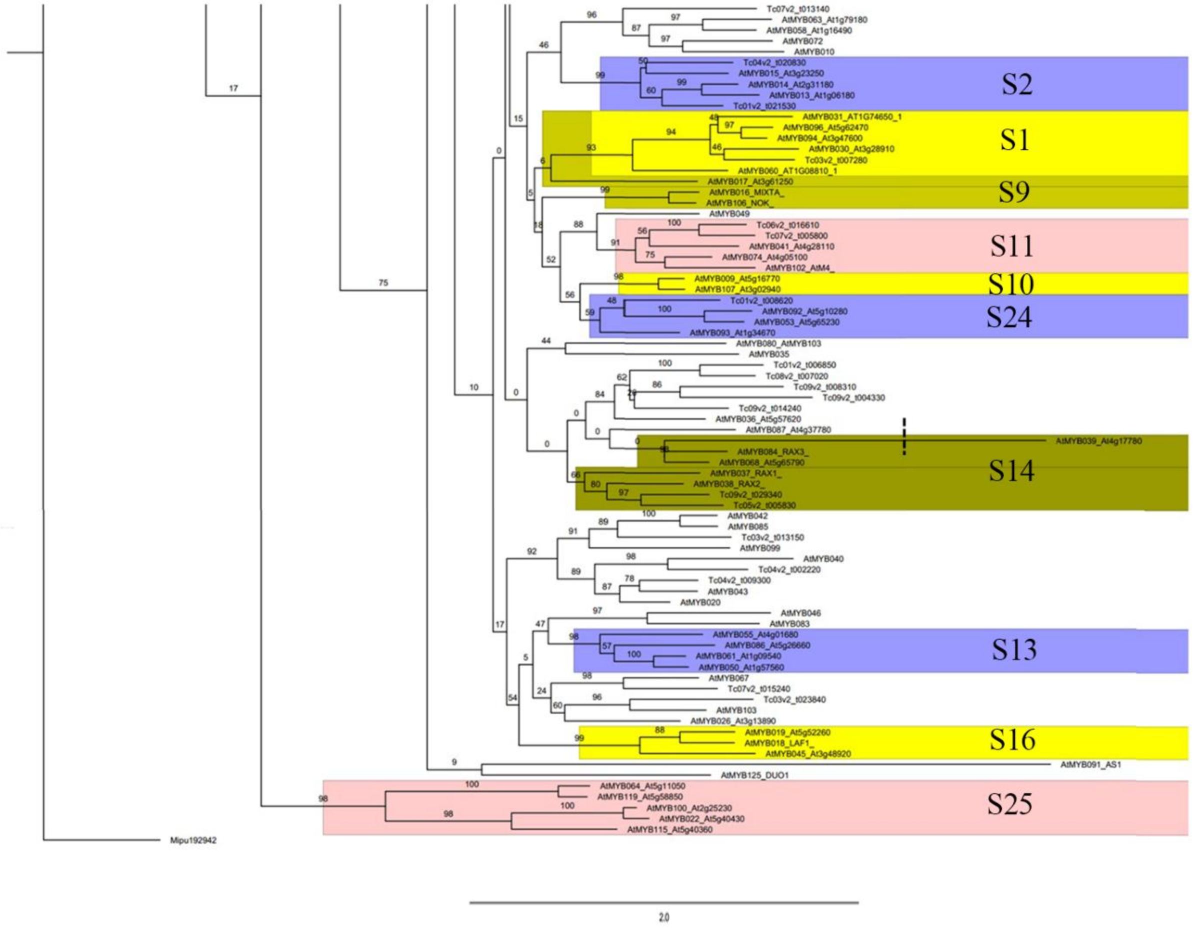

Figure S8. Schematic representation of the relationships between the different R2R3-MYB subgroups for Cacao and Arabidopsis. Analysis inferred using RAxML with up to 1000 bootstraps with CDS full length MYB sequences. The subgroups were designated as previously reported[1]. MYB Mipu192942 from *Micromonas Pusilla* was used as outgroup. Colored classification was based in[19].

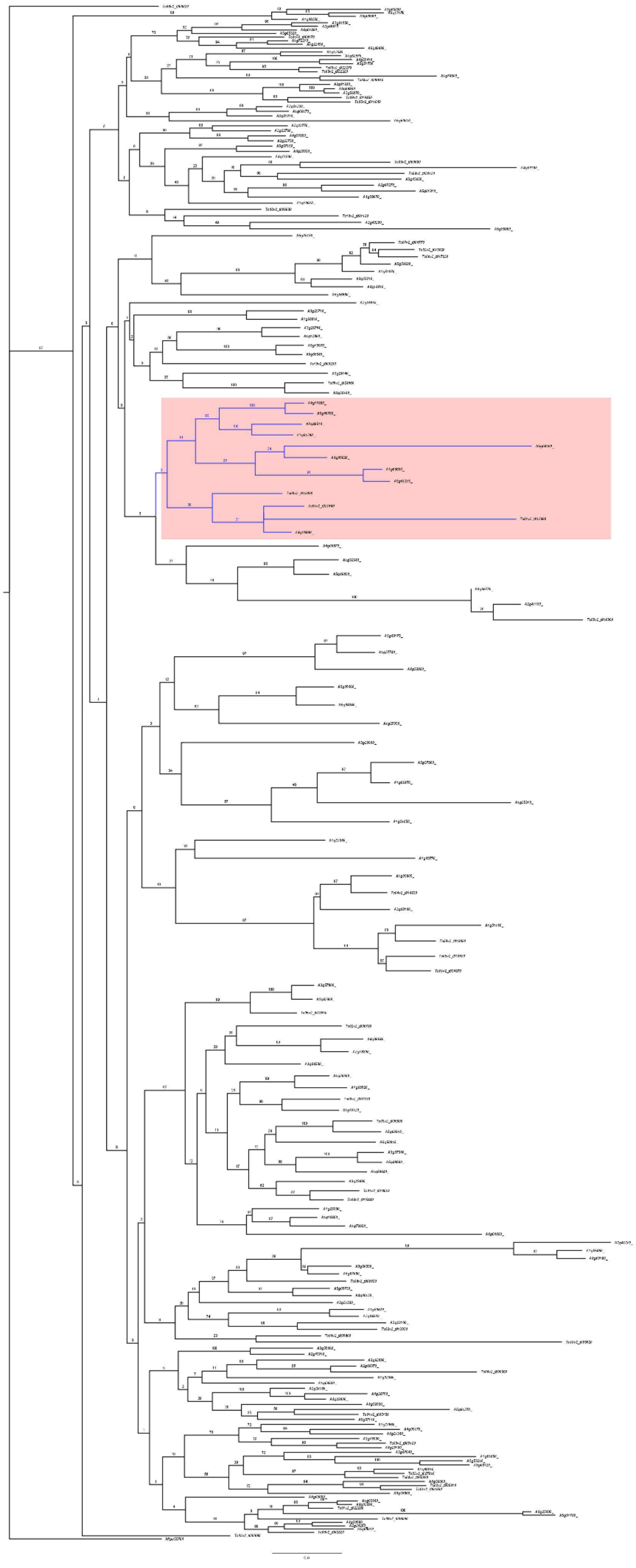

Figure S9. Schematic representation of the relationships between the different bHLH genes for Cacao and Arabidopsis. Analysis inferred using RAxML with up to 1000 bootstraps and CDS full length bHLH sequences. The subgroups were designated as previously reported[2]. The bHLH Mipu55764 from *Micromonas Pusilla* was used as outgroup. Cluster with ATTT8, AtEGL3 and AtGL1 is highlighted in pink.

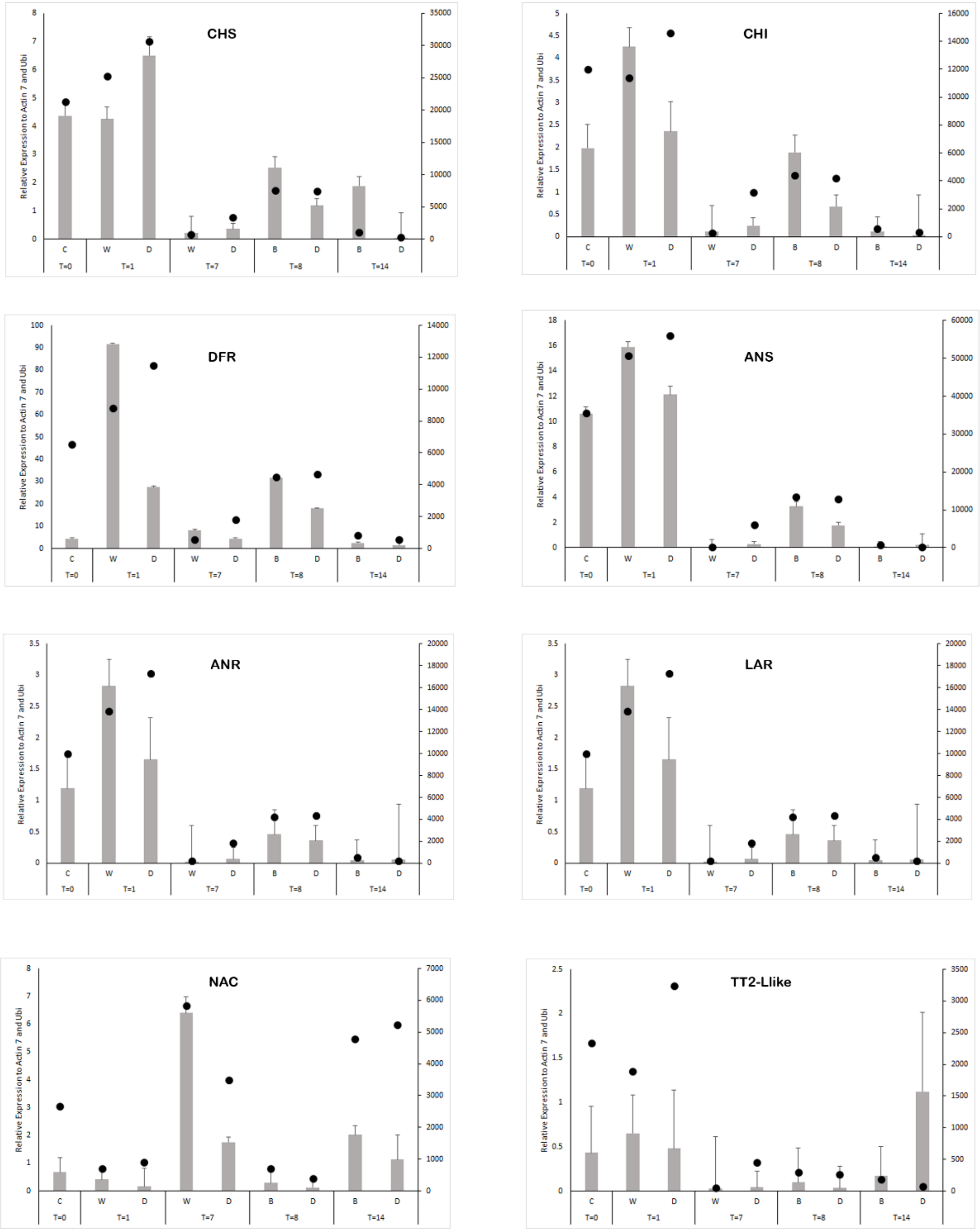

Figure S10. qRT-PCR analysis of differentially expressed genes in cacao cell suspensions. Transcript levels and qRT-PCR results of 8 randomly selected genes from RNA-sequencing. The left y-axis shows the relative gene expression levels analyzed by qPCR (gray columns). The right y-axis indicates the corresponding expression data of RNA-seq (black dots). The x-axis represents the time (days) of light/dark exposure. Bars represent SD (n = 3).
